# Nature of Anti‐Dissipative High‐Energy Excited States in Quaterpyridine‐Bridged Ruthenium Complexes

**DOI:** 10.1002/anie.202507738

**Published:** 2025-06-30

**Authors:** Noémie Chantry, Agustina Cotic, Simon De Kreijger, Riccardo Di Forti, Benjamin Elias, Ludovic Troian‐Gautier, Alejandro Cadranel

**Affiliations:** ^1^ Université catholique de Louvain (UCLouvain) Institut de la Matière Condensée et des Nanosciences (IMCN) Molecular Chemistry Materials and Catalysis (MOST) Place Louis Pasteur 1, bte L4.01.02 Louvain‐la‐Neuve 1348 Belgium; ^2^ Universidad de Buenos Aires Facultad de Ciencias Exactas y Naturales Departamento de Química Inorgánica Analítica y Química Física Pabellón 2, Ciudad Universitaria Buenos Aires C1428EHA Argentina; ^3^ CONICET − Universidad de Buenos Aires Instituto de Química‐Física de Materiales Medio Ambiente y Energía (INQUIMAE) Pabellón 2, Ciudad Universitaria Buenos Aires C1428EHA Argentina; ^4^ Wel Research Institute Avenue Pasteur 6 Wavre 1300 Belgium; ^5^ Physical Chemistry I Friedrich‐Alexander‐Universität Erlangen‐Nürnberg (FAU) Egerlandstr. 3 91058 Erlangen Germany; ^6^ Interdisciplinary Center for Molecular Materials Friedrich‐Alexander‐Universität Erlangen‐Nürnberg (FAU) Egerlandstr. 3 91058 Erlangen Germany

**Keywords:** Anti‐Kasha, Dissipation, ILET, Solar energy conversion, Ultrafast spectroscopy

## Abstract

High‐energy excited states with slow rates for internal conversion to the lowest‐energy excited state are prone to be intercepted before they dissipate energy to the medium. In a previous report, oligomeric Ru(II) photosensitizers bearing a bridging 2,2′:5′,3″:6″,2^‴^–quaterpyridine scaffold showed promising anti‐dissipative behavior in photoinduced electron transfer reactivity. In here, a range of electron accepting and electron donating substituents were incorporated on the ancillary 2,2′‐bipyridine ligands to modulate the excited‐state dynamics. This allowed to unambiguously identify the nature of high‐energy excited states and derive design guidelines for the achievement of anti‐dissipative behavior in oligomeric Ru(II) polypyridines relevant for solar fuels production and photoredox catalysis.

## Graphical abstract

Upon photoexcitation, *meta*‐connected quaterpyridine‐bridged Ru complexes undergo fast inter‐ligand electron transfer like typical chromophores, but unusually slow intra‐ligand electron transfer within bridge‐centered MLCT states. The energy saved was exploited for photoreductions and photooxidations with model electron donors and acceptors, respectively. Design guidelines involving ancillary‐ligand substitution and bridge topology were derived.

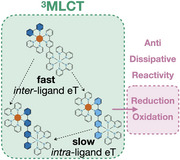

## Introduction

Utilization of high‐energy (HE) excited states for useful photochemistry^[^ ^1^, ^2^ ^]^ is such an enormous challenge that nature itself, after millions of years of evolution, has not mastered it. In fact, in natural photosynthesis, HE excited states dissipate 20%–30% of their energy through vibrational relaxation (VR) and internal conversion (IC), leading to the lowest‐energy (LE) excited state from which chemical reactions take place.^[^ ^3^ ^]^ Anti‐dissipative strategies were postulated to prevent these energy losses or slow down the timescale at which they take place, thereby allowing to trap high‐energy excited states competent for energy demanding bimolecular reactions.^[^ ^4^ ^]^

**Figure 1:**
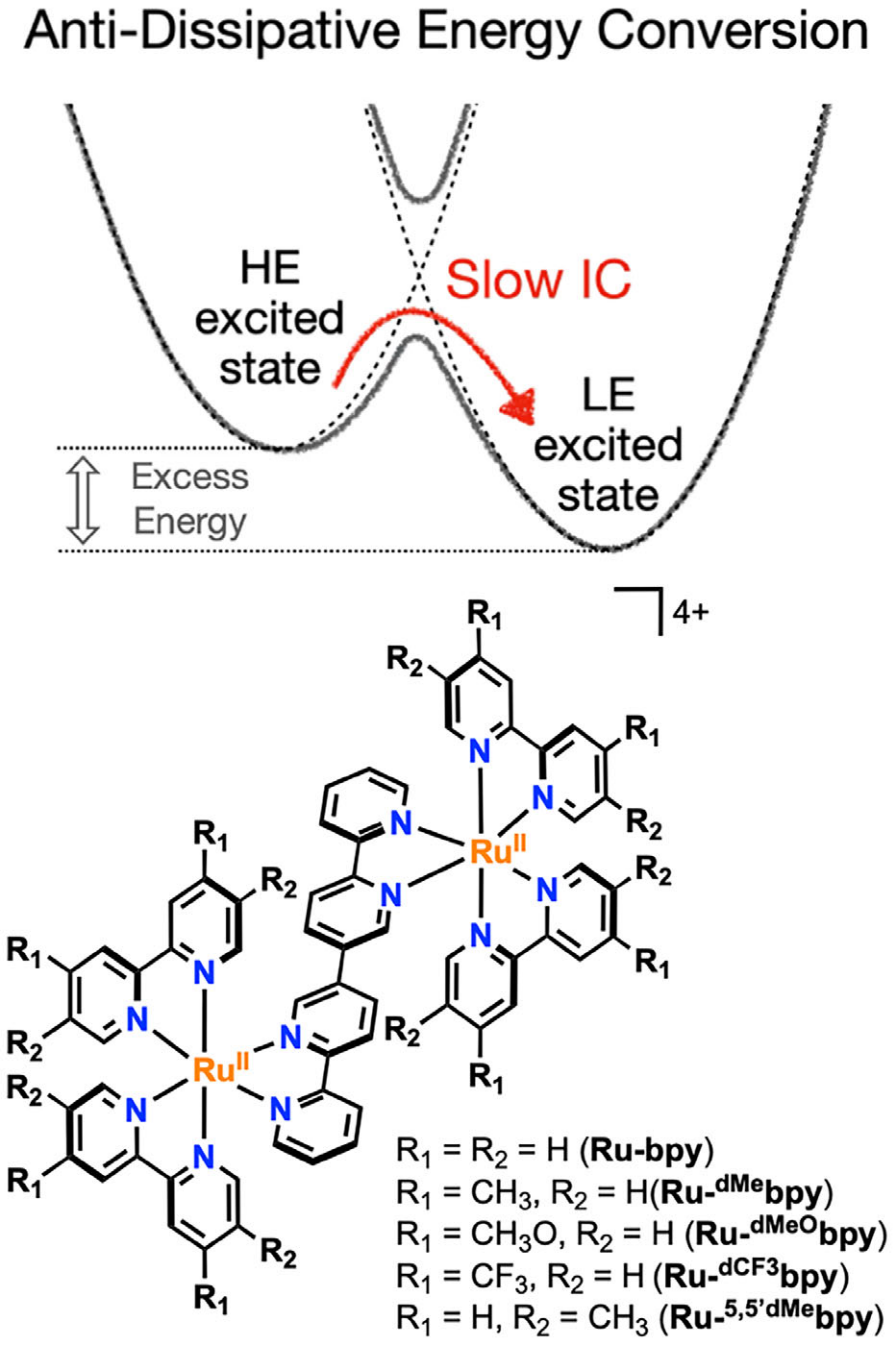
Schematic representation of anti‐dissipative (anti‐Kasha) scenarios (top) and structures and acronyms of the dinuclear complexes studied herein (bottom).

There are three key aspects in the rational design of anti‐dissipative strategies. First, the HE excited‐state lifetime needs to be compatible with the timescale of bimolecular or long‐range reactivity.^[^ ^5^ ^]^ To this end, direct decay of HE states to the ground state needs to be slow. Next, IC/VR processes that convert HE in LE states must be inhibited. This implies circumventing Kasha's rule, which often results from strong electronic coupling between the different excited states leading to negligible barriers for IC/VR dissipative pathways (Figure 1). As such, the second key aspect is to develop scenarios where the excited‐state electronic coupling is weak. This induces non‐negligible kinetic barriers that mediate the IC and VR processes, leading to anti‐Kasha photochemistry and anti‐dissipative energy conversion schemes.^[^ ^4^, ^6^, ^7^, ^8^ ^]^ Third, tuning the energy difference between HE and LE excited states defines the amount of energy that can be saved. These three aspects are intertwined, since the energy difference and electronic coupling between HE and LE states are intimately linked, as is the HE excited‐state lifetime that depends on the energy difference and electronic coupling with the LE state and the ground state. Control over all these aspects can be exerted only if the precise nature of HE states is identified.

Previous work from our groups focusing on binuclear and trinuclear Ru(II) photosensitizers has highlighted that an anti‐dissipative behavior could be achieved by carefully controlling the topology of the ligand bridging the different Ru(II) centers, reaching drastically different electronic coupling scenarios. All‐*meta* (5,5′) connections led to photosensitizers that exhibited an anti‐Kasha behavior (Figure 1) while photosensitizers with *para* (4,4′) connections behaved like conventional [Ru(bpy)_3_]^2+^ photosensitizers.^[^ ^2^, ^9^ ^]^ Using that approach, the co‐existence of two metal‐to‐ligand charge transfer (MLCT) excited states separated by 800 cm^−1^ could be spectroscopically observed. Lifetimes of 515 and 16 ns for the LE‐ and HE‐^3^MLCT excited states, respectively, were determined by transient absorption spectroscopy for the binuclear photosensitizers, whereas they were 312 ns (LE) and 9 ns (HE) for the trinuclear analogue. Proof‐of‐concept excited‐state quenching experiments with a sacrificial electron donor demonstrated that both HE and LE states undergo bimolecular electron transfer. We already reported an initial hypothesis, substantiated in the different spectral signatures obtained for HE and LE states upon ultrafast transient‐absorption spectroscopy, which involved bridge‐centered LE‐^3^MLCT states, and HE‐^3^MLCT states located on ancillary bpy ligands.^[^ ^4^ ^]^

Building on that discovery, we sought to investigate how electron donating or withdrawing substituents on the ancillary 2,2′‐bipyridine ligands would impact the anti‐dissipative behavior, while keeping the weak electronic coupling scenario introduced by the bridging 2,2′:5′,3″:6″,2^‴^‐quaterpyridine (L_m_) (Figure 1). These changes allow to systematically tune the energy level of the ancillary ligand vis‐à‐vis the bridging ligand. Using classical spectroscopy methods (steady state absorption, photoluminescence, and time‐resolved photoluminescence), (spectro)electrochemistry as well as more advanced nanosecond and femtosecond transient absorption spectroscopy (nsTAS and fsTAS, respectively), we identified the nature of HE‐^3^MLCT states and derive design rules for anti‐dissipative chromophores.

## Results and Discussion

### Synthesis

The L_m_ bridging ligand was synthesized as previously reported using the nickel‐catalyzed homocoupling of 5‐bromo‐2,2′‐bipyridine.^[^ ^10^ ^]^ The substituted derivatives of [Ru(LL)_2_Cl_2_], where LL is 4,4′‐dimethyl‐2,2′‐bipyridine, 5,5′‐dimethyl‐2,2′‐bipyridine, 4,4′‐dimethoxy‐2,2′‐bipyridine or 4,4′‐bis(trifluoromethyl)‐2,2′‐bipyridine were synthesized in two steps. The first step involved the synthesis of Ru(COD) from RuCl_3_ and cyclooctadiene (COD) in ethanol, whereas the second step involved the reaction of Ru(COD) with two LL substituted ligand in dichlorobenzene at 180 °C for 2 h.^[^ ^11^, ^12^ ^]^ The compounds were then isolated by precipitation and used without any further purification. For the synthesis of the bimetallic photosensitizers, an excess of [Ru(LL)_2_Cl_2_] was mixed with the L_m_ ligand in an ethylene glycol/water mixture in the presence of silver nitrate and heated at 180 °C for 1 h via microwave irradiation. The complexes were purified on silica gel column and when necessary, by a complementary LH20 size exclusion chromatography. The photosensitizers were finally isolated as the hexafluorophosphate salts after ion metathesis and characterized by ^1^H‐NMR (Figures S1–S4) and high‐resolution mass spectrometry. The detailed procedures are provided in the Supporting Information.

### Electrochemistry

**Figure 2:**
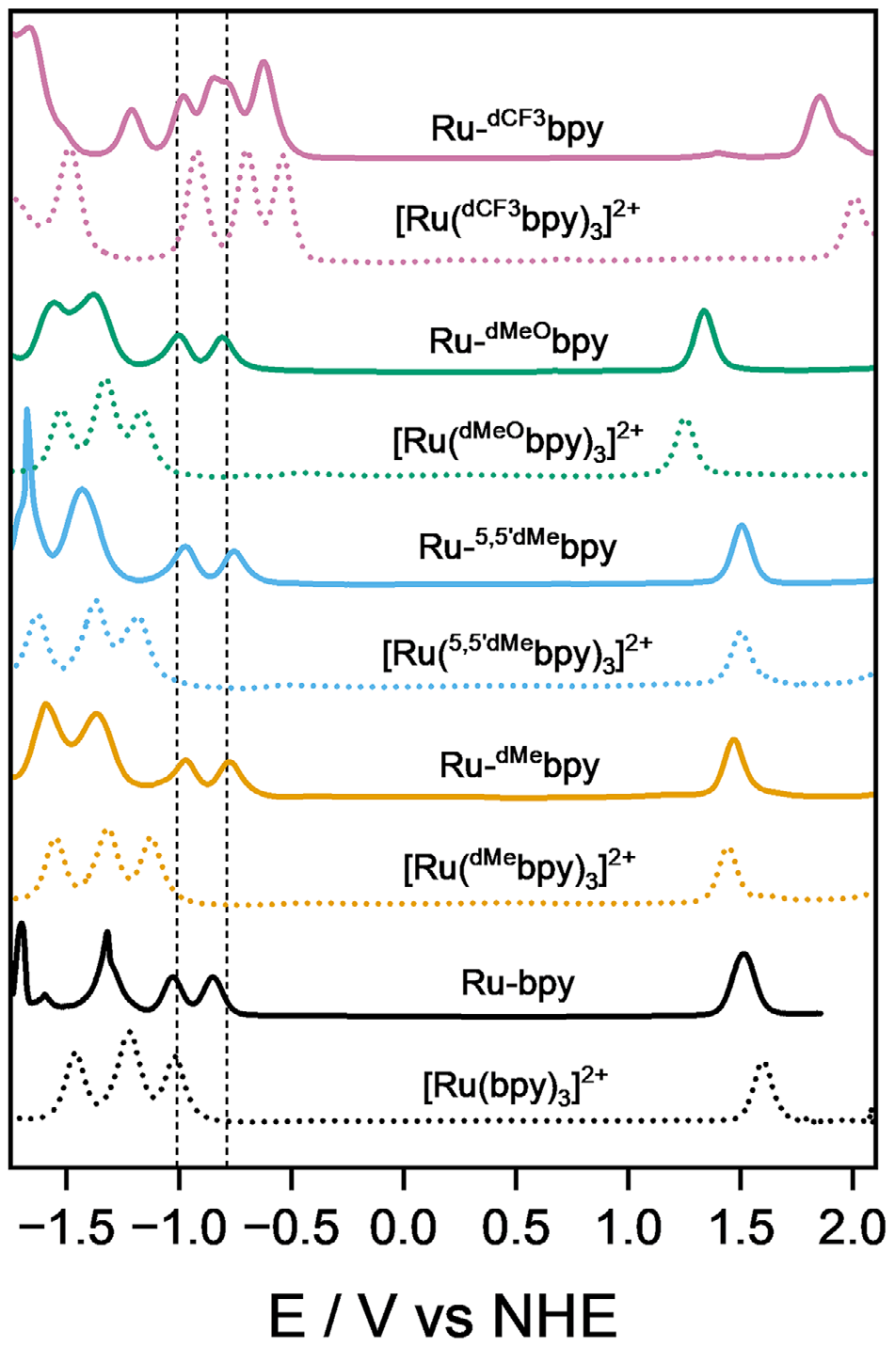
Differential pulse voltammetry of Ru‐bpy (black), Ru‐^dMe^bpy (orange), Ru‐^5,5′‐dMe^bpy (cyan), Ru‐^dMeO^bpy (green), and Ru‐^dCF3^bpy (magenta) (all solid curves), and all the corresponding homoleptic complexes (all dotted curves). L_m_‐centered reductions at nearly constant potentials are indicated with vertical dashed lines. All the measurements were carried out in acetonitrile with 100 mM TBAPF_6_ as supporting electrolyte.

The electrochemical behavior of all photosensitizers was investigated in acetonitrile containing 0.1 M TBAPF_6_ supporting electrolyte. All photosensitizers exhibited a single two‐electron oxidation wave at positive potentials (Figure 2). These potentials span from 1.33 V versus NHE for Ru‐^dMeO^bpy, including the strongly electron‐donating methoxy substituents, to 1.85 V versus NHE for Ru‐^dCF3^bpy, bearing the strongly electron‐accepting trifluoromethyl groups. This oxidation wave was attributed to the simultaneous oxidation of both metal centers and confirmed that the poor electronic coupling in the ground state afforded by the L_m_ bridge persisted for all compounds despite ancillary ligand substitution. Importantly, integration of the area under this two‐electron wave served as an internal reference for the analysis of the rich reductive electrochemistry, which was crucial to identify the nature of the different charge‐transfer excited states. Previous studies on homoleptic derivatives of [Ru(bpy)_3_]^2+^ have shown that the reduction potential of the bpy ligand is influenced by the presence of substituents.^[^ ^13^, ^14^, ^15^, ^16^ ^]^ For Ru‐^dCF3^bpy, the strong electron accepting character facilitated the first reduction on the ancillary ^dCF3^bpy at −0.62 V versus NHE. This wave integrated for two electrons, consistent with a poor electronic coupling scenario where two ^dCF3^bpy, one attached to each Ru ion, are simultaneously reduced. This signal was followed by closely spaced waves that included reductions on the remaining ancillary ligands and the bridge. All other photosensitizers exhibited two identical, one‐electron reduction waves at nearly constant values of −0.80 ± 0.05 and −1.00 ± 0.03 V versus NHE, assigned to two successive reduction processes on the bridge, based on the integration and on the comparison with reference homoleptic complexes (Figure 2). These were followed by multi‐electronic waves below −1.3 V versus NHE, most likely corresponding to the ancillary ligands, shifting to more negative potentials for electron donating substituents (Table 1). The case of Ru‐bpy was more intricate. Indeed, while it was clear that the first reduction was bridge based, determining the exact nature of the second reduction process was less evident than for the other complexes as it occurred at the same potential as the first reduction of the corresponding homoleptic [Ru(bpy)_3_]^2+^. However, based on the identical one‐electron integration for this second reduction wave across the whole range of complexes, we safely concluded that it was also bridge based for Ru‐bpy. For this compound, and for Ru‐^dMe^bpy, Ru‐^5,5′dMe^bpy, and Ru‐^dMeO^bpy, the energy difference between the first two reductions was nearly constant and on average ≈200 mV (≈1600 cm^−1^). In these complexes, the two successive reduction events on the bridging ligand shifted the reduction potential of the ancillary bpy to more negative potentials relative to the homoleptic references, most likely due to coulombic interactions.

**Table 1:** Redox potentials of Ru(II) complexes. All the measurements were carried out in acetonitrile with 100 mM TBAPF_6_ as supporting electrolyte.

	E [V vs NHE] (e^–^ equivalents)					
Compounds	Ru^III^/Ru^II^	L_m_/L_m_^–^	L_m_^–^/L_m_^2–^	^R^bpy/^R^bpy^–^		
[Ru(bpy)_3_]^2+^	1.60 (1)	−	−	−1.02 (1)	−1.22 (1)	−1.46 (1)
Ru‐bpy	1.51 (2)	−0.85 (1)	−1.03 (1)	−1.32 (2)	n.d.	
[Ru(^dMe^bpy)_3_]^2+^	1.44 (1)	−	−	−1.12 (1)	−1.31 (1)	−1.55 (1)
Ru‐^dMe^bpy	1.47 (2)	−0.78 (1)	−0.97 (1)	−1.38 (2)	−1.60 (2)	
[Ru(^5,5′dMe^bpy)_3_]^2+^	1.50 (1)	−	−	−1.18 (1)	−1.37 (1)	−1.63 (1)
Ru‐^5,5′dMe^bpy	1.51 (2)	−0.76 (1)	−0.97 (1)	−1.43 (2)	−1.68 (2)	
[Ru(^dMeO^bpy)_3_]^2+^	1.25 (1)	−	−	−1.16 (1)	−1.33 (1)	−1.53 (1)
Ru‐^dMeO^bpy	1.33 (2)	−0.81 (1)	−1.00 (1)	−1.39 (2)	−1.56 (2)	
[Ru(^dCF3^bpy)_3_]^2+^	2.01 (1)	−	−	−0.54 (1)	−0.69 (1)	−0.91(1)
Ru‐^dCF3^bpy	1.85 (2)	−0.98 (1)	−1.21 (1)	−0.62 (2)	−0.82 (2)	

### Absorption Spectroscopy and Spectroelectrochemistry

**Figure 3:**
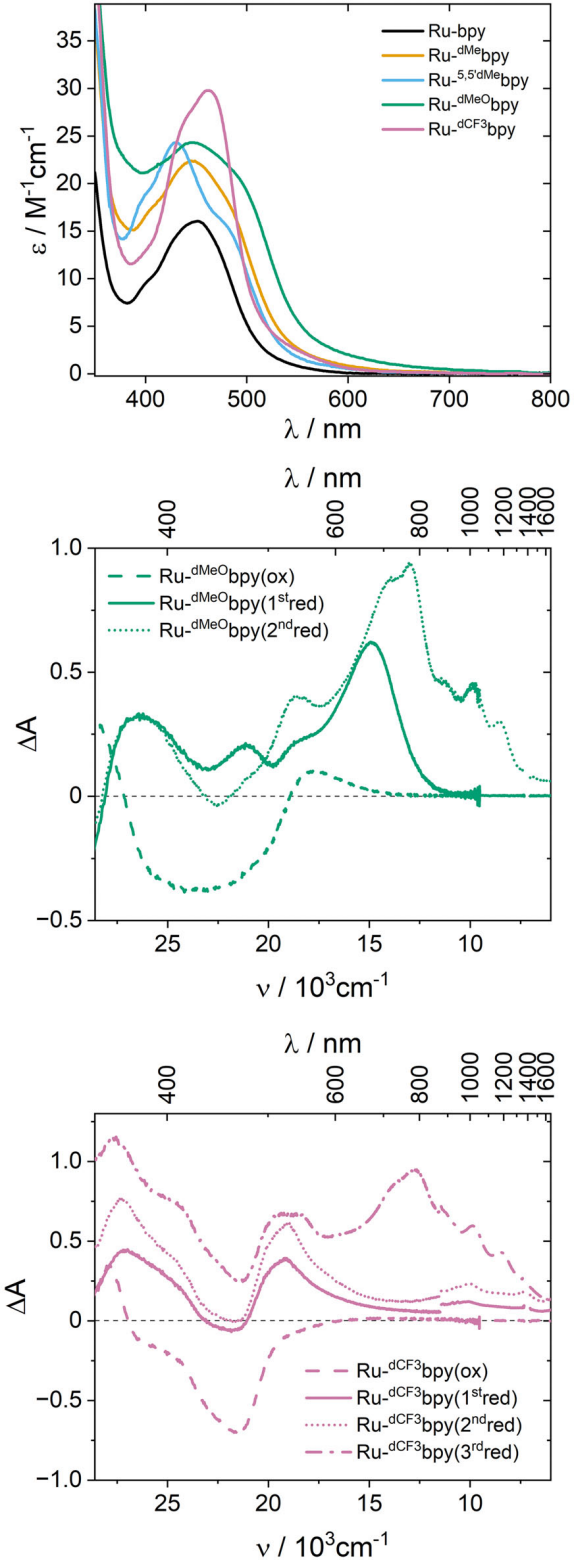
Top: absorption spectra of Ru‐bpy (black), Ru‐^dMe^bpy (orange), Ru‐^5,5′‐dMe^bpy (cyan), Ru‐^dMeO^bpy (green), and Ru‐^dCF3^bpy (magenta) in acetonitrile. Middle: Differential spectra obtained upon oxidation (dashed), first reduction (solid) and second reduction (dotted) of Ru‐^dMeO^bpy in acetonitrile with 100 mM TBAPF_6_ as supporting electrolyte. Bottom: Differential spectra obtained upon oxidation (dashed), first reduction (solid), second reduction (dotted), and third reduction (dash‐dotted) of Ru‐^dCF3^bpy in acetonitrile with 100 mM TBAPF_6_ as supporting electrolyte.

Ground‐state UV–visible absorption spectra were recorded in acetonitrile (Figure 3). They presented absorption features typical for ruthenium polypyridines, with narrow bands in the UV region attributed to ligand centered (LC) transitions and broad bands in the visible attributed to metal‐to‐ligand charge transfer (MLCT) transitions. The addition of electron donating substituents led to only small changes in the broadness and position of the UV–visible absorptions, with the methoxy substituent extending absorption toward longer wavelengths. The main difference was observed when the CF_3_ substituents were incorporated, which led to an increase in molar absorption coefficient and the recovery of a MLCT transition that was more akin to prototypical [Ru(bpy)_3_]^2+^.

Spectroelectrochemical experiments provided further details related to the nature of the different redox processes. Oxidations proceeded cleanly for all photosensitizers, with disappearance of MLCT absorptions and absence of any significant NIR signals that would have corresponded to intervalence charge transfer (IVCT) transitions (Figures 3 and S5), consistent with the poor electronic coupling. Like previously observed for Ru‐bpy,^[^ ^4^ ^]^ the first reduction of Ru‐^dMe^bpy, Ru‐^5,5′dMe^bpy, and Ru‐^dMeO^bpy, led to the appearance of typical polypyridine radical anion signatures around 350–400 and 500 nm, accompanied by an intense band around 660 nm (Figures 3 and S5). These differential spectra are remarkably similar to that recently reported for a {Ir(L_m_)Re} complex,^[^ ^17^ ^]^ where, crucially, the only redox‐active ligand is L_m_. This confirms that the first reduction produced the mono‐reduced bridging ligand L_m_^•–^ in the photosensitizers studied herein, except for Ru‐^dCF3^bpy. Indeed, in this latter, the bpy^•–^ hallmarks around 370 and 500 nm were observed, but the signal at 660 nm was absent (Figure 3). In this case, the first reduction was centered on the ancillary ^dCF3^bpy, as stated above.

For Ru‐^dMe^bpy, Ru‐^5,5′dMe^bpy, and Ru‐^dMeO^bpy, the second reduction afforded remarkably similar differential spectra governed by an intense positive band around 800 nm, accompanied by weaker absorptions toward the NIR and toward the visible (Figures 3 and S5). These transitions are in line with those recently documented for Ru‐bpy^[^ ^4^ ^]^ and the {Ir(L_m_)Re} compounds^[^ ^17^ ^]^ upon two‐electron reduction. Therefore, the second reduction in Ru‐bpy, Ru‐^dMe^bpy, Ru‐^5,5′dMe^bpy, and Ru‐^dMeO^bpy produced the doubly‐reduced bridge. In contrast, for Ru‐^dCF3^bpy the 800 nm band was absent for the second reduction, which therefore corresponded to the reduction of the remaining neutral ancillary ^dCF3^bpy. It was only upon the third reduction process that a band consistent with the bridge reduction occurred for Ru‐^dCF3^bpy (Figure 3). These assignments agree with the interpretation of electrochemical experiments based upon current integration (vide supra) and provide a useful handle for the identification of the different excited states observed in transient‐absorption spectroscopy.

### Steady‐State and Time‐Resolved Emission Spectroscopy

**Figure 4:**
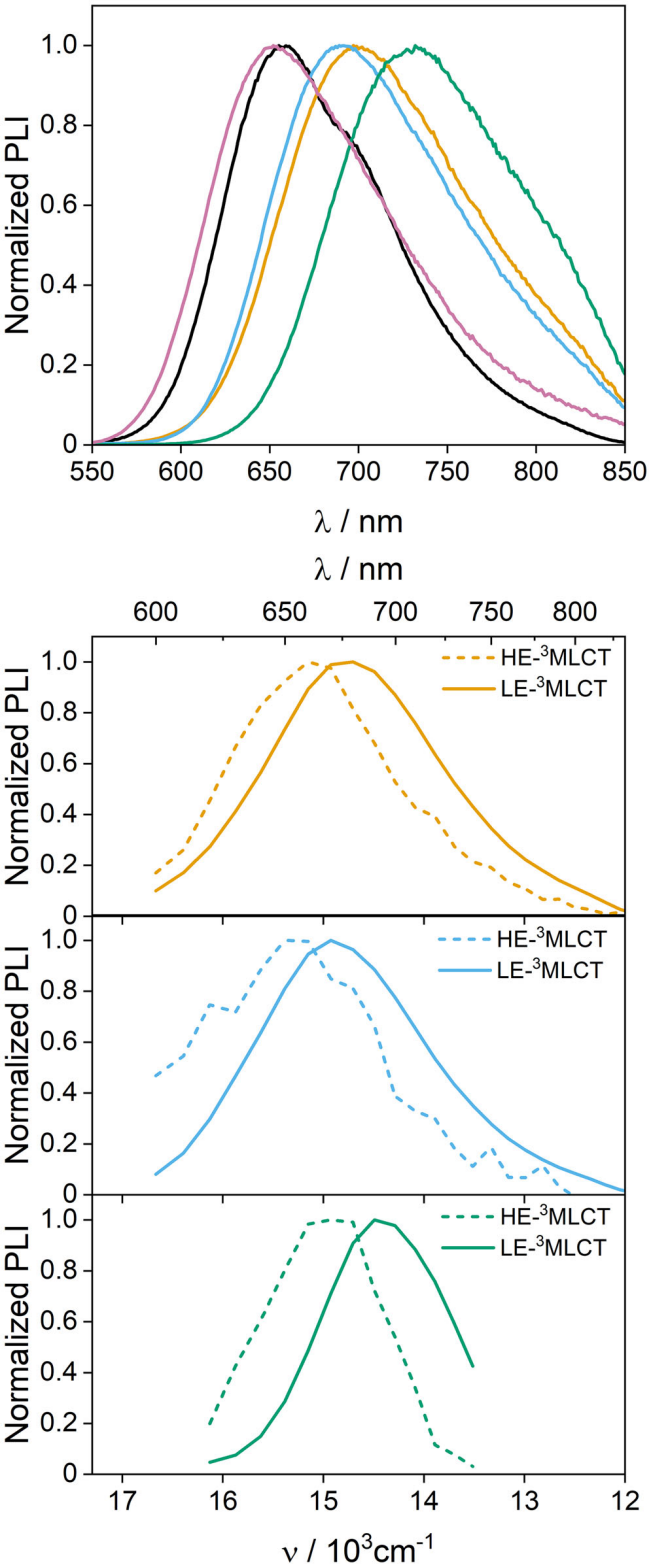
Top: Steady‐state (corrected) emission of Ru‐bpy (black), Ru‐^dMe^bpy (orange), Ru‐^5,5′‐dMe^bpy (cyan), Ru‐^dMeO^bpy (green), and Ru‐^dCF3^bpy (magenta) in argon‐purged acetonitrile at room temperature. Bottom: TRES (uncorrected) of Ru‐^dMe^bpy (orange), Ru‐^5,5′‐dMe^bpy (cyan), and Ru‐^dMeO^bpy (green) in acetonitrile at room temperature.

Steady‐state photoluminescence was observed for all photosensitizers in argon‐purged acetonitrile at room temperature. Spectra (corrected for the PMT's response) are shown in Figure 4 and relevant data are collected in Table 2. No excitation‐wavelength dependence neither on the emission band shape nor on the emission wavelength was observed (Figure S6). Relative to Ru‐bpy, substitution with electron donating groups on the ancillary ligands led to a red shift of the emission maxima. This shift was stronger for Ru‐^dMeO^bpy, with a band peaking at 732 nm, in line with a smaller HOMO‐LUMO gap, as expected based on the UV–visible absorption spectra and the electrochemical data (Figure 3 and Table 1).^[^ ^18^ ^]^ This was consistent with a LE excited state being an emissive ^3^MLCT localized on the bridging ligand, and largely dominating for Ru‐bpy, Ru‐^dMe^bpy, Ru‐^5,5′dMe^bpy, and Ru‐^dMeO^bpy. Strikingly, for Ru‐^CF3^bpy, the maximum was blue‐shifted but still close to that of Ru‐bpy. This stemmed from a LE‐^3^MLCT localized on the ancillary dCF_3_‐bpy ligand, where the more positive metal‐centered oxidation potential was compensated by the less negative reduction of the ancillary ligands (Table 1). Indeed, the emission maximum was located at 652 nm for Ru‐^dCF3^bpy, close to the one of [Ru(^dCF3^bpy)_3_]^2+^ reported in the literature at 633 nm.^[^ ^14^ ^]^ Franck–Condon line shape analysis of the 77K emission spectra in butyronitrile (Figure S7) resulted in *E*_00_ values for the dominant LE‐^3^MLCT between 15200 to 16400 cm^−1^, ordered according to the room‐temperature emission of each compound (Table 2). Importantly, Huang–Rhys factors, determined from the steady‐state photoluminescence recorded at 77K, are smaller for Ru‐bpy, Ru‐^dMe^bpy, Ru‐^5,5′dMe^bpy, and Ru‐^dMeO^bpy (Table S1) than those determined for the corresponding monometallic references, revealing a less distorted, and thus more delocalized nature for LE‐^3^MLCT in the bimetallic complexes and consistent with a bridging‐ligand centered assignment. In contrast, for Ru‐^CF3^bpy, Huang–Rhys factors are very similar to those of [Ru(^dCF3^bpy)_3_]^2+^, suggesting a very similar nature of LE‐^3^MLCT in both systems, centered on ^dCF3^bpy.

**Table 2:** Excited‐state properties recorded in argon‐purged solutions.

	LE‐^3^MLCT					HE‐^3^MLCT				
Compound	*λ*_em_ (nm) (Φ_em_)	*τ*_TCSPC_/τ_TA_ (ns)	*E*_00_ (cm^−1^)	*E*_red_*(LE) (V)^c)^	*E*_ox_*(LE) (V)^c)^	*τ*_TCSPC_/*τ*_TA_ (ns)	*E*_00_ (cm^−1^)	*E*_red_*(HE) (V)^c)^	*E*_ox_*(HE) (V)^c)^	*ΔE*(HE–LE) (eV)^d)^
Ru‐^dCF3^bpy	652 (0.070)	927/890	16110	1.31	−0.08	−	−	−	−	−
Ru‐bpy	655 (0.034)	515^a)^	16400^b)^	1.12	−0.46	16^a)^	17380	1.12	−0.58	0.12
Ru‐^dMe^bpy	697 (0.019)	276/274	15890	1.13	−0.47	18/18	16350	1.13	−0.52	0.06
Ru‐^5,5′dMe^bpy	689 (0.033)	269/356	15740	1.13	−0.42	4/11	16200	1.13	−0.47	0.06
Ru‐^dMeO^bpy	732 (0.005)	94/92	15240	1.02	−0.50	14/44	15670	1.02	−0.55	0.05

^a)^ From ref.[4]

^b)^ Obtained from the value for LE‐^3^MLCT plus *ΔE*(HE–LE).

^c)^ V versus NHE.

^d)^ Energy differences were obtained from the non‐corrected emission resolved by TRES.

Emission lifetimes under inert atmosphere were obtained from time‐correlated single‐photon counting (TCSPC) measurements at the respective emission maxima using 460 nm excitation (Figure S8). Biexponential decays were observed for Ru‐^dMe^bpy, Ru‐^5,5′dMe^bpy, and Ru‐^dMeO^bpy, with lifetimes of 18 and 276 ns, 4 and 269 ns, and 14 and 94 ns, respectively. They were ascribed to HE and LE states, respectively, like in Ru‐bpy. In contrast, Ru‐^dCF3^bpy behaved as a classic ruthenium(II) polypyridine complex with a monoexponential decay lifetime of 927 ns. Time‐resolved emission spectroscopy (TRES) was utilized to gain deeper insights into the relative contributions of the HE states to the overall emission. Target analysis of the TCSPC data revealed HE‐ and LE‐^3^MLCT emission maxima (uncorrected) at 650 and 670 nm, 650 and 670 nm, and 670 and 690 nm, for Ru‐^dMe^bpy, Ru‐^5,5′dMe^bpy, and Ru‐^dMeO^bpy, respectively, which compared well with those reported for Ru‐bpy, i.e., 620 and 660 nm.^[^ ^4^ ^]^ Using these values, spectroscopic energy differences between HE‐ and LE‐^3^MLCT states (*ΔE*(HE–LE)) of 980, 460, 460 and 430 cm^−1^ (0.12, 0.06, 0.06 and 0.05 eV), for Ru‐bpy, Ru‐^dMe^bpy, Ru‐^5,5′dMe^bpy, and Ru‐^dMeO^bpy could be estimated, respectively (Table 2). While variations were slight, electron‐donating substituents on ancillary ligands seem overall to decrease *ΔE*(HE–LE). For Ru‐^dMe^bpy, Ru‐^5,5′dMe^bpy, and Ru‐^dMeO^bpy, HE‐^3^MLCT emission intensity was around 10% relative to that for LE‐^3^MLCT. This is consistent with steady‐state emission spectra, both at room temperature and 77K, fully dominated by LE‐^3^MLCT.

### Transient Absorption Spectroscopy

The nanosecond timescale dynamics of the different complexes in acetonitrile was investigated using nsTAS with 460 nm excitation at room temperature under inert atmosphere. For Ru‐^dMe^bpy, Ru‐^5,5′dMe^bpy, and Ru‐^dMeO^bpy, inspection of the kinetic traces between 500 and 515 nm revealed a rise in the first 30 ns, before the signal decayed to zero indicating ground‐state recovery (Figures S9–S11). These results showed that at least two excited states participated in the nanosecond deactivation cascade. The lifetimes and species‐associated differential spectra for both states of Ru‐^dMe^bpy, Ru‐^5,5′dMe^bpy, and Ru‐^dMeO^bpy were extracted using a target analysis procedure, considering a model previously used for Ru‐bpy (Figure S13).^[^ ^4^ ^]^ The longer‐lived species, LE‐^3^MLCT, displayed a lifetime ranging from 92 to 356 ns (Table 2) with an associated differential spectrum showing a broad absorption across the visible range.^[^ ^4^, ^9^, ^17^ ^]^ The shorter‐lived species, HE‐^3^MLCT, exhibited a lifetime of 11–44 ns with an associated differential absorption pattern similar to the classical [Ru(bpy)_3_]^2+^. In short, these complexes behave like Ru‐bpy.^[^ ^4^ ^]^ In contrast, a mono‐exponential fit was sufficient for Ru‐^dCF3^bpy, with a lifetime of 890 ns and a differential spectrum showing the typical features of [Ru(^dCF3^bpy)_3_]^2+^ (Figure S12). All HE‐ and LE‐^3^MLCT lifetimes determined using nsTAS were consistent with those derived from TCSPC within experimental errors.

**Figure 5:**
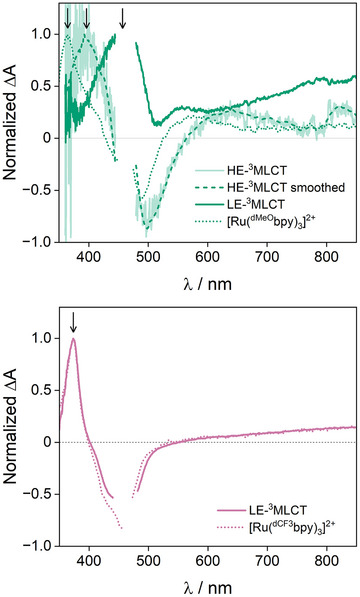
Species‐associated differential spectra obtained upon nsTAS with 460 nm excitation of Ru‐^dMeO^bpy (top) and Ru‐^dCF3^bpy (bottom) and their respective monometallic references in acetonitrile at room temperature under inert atmosphere. Arrows indicate radical anion signals for each excited state. For HE‐^3^MLCT, a smoothed spectrum is also shown for clarity (see experimental details in the Supporting Information).

At this point, gathering results from electrochemistry, spectroelectrochemistry, photoluminescence spectroscopy, and nsTAS in the differently substituted photosensitizers enabled an accurate description of the exact nature of LE‐ and HE‐^3^MLCT. For Ru‐^dCF3^bpy, thanks to the strong electron accepting substituents, LE‐^3^MLCT included an excited electron on ^dCF3^bpy, and was therefore labeled LE‐^3^MLCT(^dCF3^bpy). In this bimetallic complex, LE‐^3^MLCT featured an intense photoinduced absorption at 373 nm ascribed to ^dCF3^bpy^•–^, exactly as in the reference [Ru(^dCF3^bpy)_3_]^2+^ (see black arrow in Figure 5). This means that the corresponding monometallic complexes are excellent models when ancillary ligand‐centered excited states in the bimetallic systems are at stake. For Ru‐bpy, Ru‐^dMe^bpy, Ru‐^5,5′dMe^bpy, and Ru‐^dMeO^bpy, LE‐^3^MLCT corresponded to an excited electron delocalized over the L_m_ bridge moiety and was thus labeled LE‐^3^MLCT(L_m_^deloc^). Their differential spectra were governed by photoinduced absorptions at 435–450 nm ascribed to L_m_^•–^ (Figures 5 and S9–S11). Here, it is useful to discuss our previously reported DFT/TD‐DFT calculations on Ru‐bpy,^[^ ^4^ ^]^ which allowed to compute the lowest‐energy triplet state LE‐^3^MLCT. The calculated electronic structure involved an excited hole essentially localized on one Ru ion, and an excited electron delocalized over the bridging ligand. It should be noted, however, that the excited electron was asymmetrically distributed, with stronger components over the region proximal to the excited hole. These calculations reinforce our assignment of LE‐^3^MLCT(L_m_^deloc^), where the “delocalized” label emphasizes the increased delocalization of the radical anion over more than simply two aromatic rings like in regular 2,2′‐bipyiridines.

While the identity of LE‐^3^MLCT was more evident, description of the precise nature of HE‐^3^MLCT required closer inspection of the species‐associated differential spectra. HE‐^3^MLCT spectra for Ru‐bpy, Ru‐^dMe^bpy, Ru‐^5,5′dMe^bpy, and Ru‐^dMeO^bpy feature photoinduced absorptions at 630, 640, 615 and 640 nm, respectively, which are absent in the corresponding monometallic references. Furthermore, the radical anion signal in HE‐^3^MLCT of the bimetallic species appeared at 410, 395, 390 and 385 nm, respectively, while photoinduced absorptions for [Ru(bpy)_3_]^2+^, [Ru(^dMe^bpy)_3_]^2+^, [Ru(^5,5′dMe^bpy)_3_]^2+^, and [Ru(^dMeO^bpy)_3_]^2+^ occurred at 374, 372, 379 and 361 nm (see black arrows in Figures 5 and S14). This means that, in contrast to the ^dCF3^bpy analogue, the HE excited electron is not localized over an ancillary ligand in the other bimetallic complexes. Precisely, HE‐^3^MLCT is, like LE‐^3^MLCT, centered on the bridging ligand. This scenario implies a HE→LE IC in the form of a slow intra‐ligand ILET (intra‐ligand electron transfer, see Equation 4), much like well‐known Ru complexes including dipyrido[3,2‐a:2′,3′‐c]phenazine (dppz) and other extended phenanthroline scaffolds which feature ILET between MLCT excited states centered on different portions of the same ligand.^[^ ^2^ ^]^

In Ru‐bpy, Ru‐^dMe^bpy, Ru‐^5,5′dMe^bpy, and Ru‐^dMeO^bpy, photoinduced π*–π* absorptions of the radical anion in HE‐^3^MLCT, at 385–410 nm, are intermediate between those in LE‐^3^MLCT at 435–450 nm and those for bpy^•–^ at 361–379 nm in the monometallic references. We considered two hypothetical origins of these spectral differences. First, we analyzed the possibility of a proximal‐distal type of description for LE and HE states, resembling the one in {Ru(dppz)} compounds.^[^ ^19^, ^20^, ^21^ ^]^ There, a phenanthroline‐based orbital, proximal to the Ru ion bearing the excited hole, and a phenazine‐based orbital, in a distal portion of the ligand, are available for the MLCT‐excited electron. Beyond the proximal‐distal location relative to the Ru ion, those moieties within dppz are structurally different. In contrast, the quaterpyridine scaffold utilized herein is symmetric, with structurally equivalent proximal and distal bpy moieties. According to this, and taking into account our previous DFT/TD‐DFT calculations mentioned above, LE‐^3^MLCT in the bimetallic complexes studied here would be a “proximal” state, and HE‐^3^MLCT would be analogous to LE‐^3^MLCT, with the bridging‐ligand radical anion delocalized, but oriented away from the excited hole. In this hypothesis, the spectral differences between LE‐ and HE‐^3^MLCT would be due to distinct Coulombic contributions from a proximal or distal excited hole, respectively, to the π* orbital spacing. Notably, under this description, once the excited hole is reduced, for example upon photoinduced electron transfer, proximal and distal MLCT states would produce fully equivalent reduced species. However, the reduced species originating from the reactions of LE and HE‐^3^MLCT with the TTA electron donor are spectrally different (vide infra). Therefore, a model considering only a proximal‐distal type of description fails to account for the observed behavior. Thus, we postulate an alternative explanation. We believe that behind the observed spectral differences between HE‐ and LE‐^3^MLCT is the fact that energy levels in aromatic chromophores are known to become closer to each other, and therefore π*–π* absorptions are expected to red‐shift upon extending the conjugation in multi‐aromatic systems.^[^ ^22^ ^]^ In this rationale, the excited electron in HE‐^3^MLCT is more localized than in LE‐^3^MLCT(L_m_^deloc^), and therefore it is labelled HE‐^3^MLCT(L_m_^loc^). These two excited states would produce, upon photoreduction of the excited hole, two different reduced species, which is consistent with our observations (vide infra). It should be noted that the “localized” label is used to emphasize the contrast with the other bridging‐ligand centered LE‐^3^MLCT(L_m_^deloc^) state, but the extent of delocalization in HE‐^3^MLCT(L_m_^loc^) is probably intermediate between LE‐^3^MLCT(L_m_^deloc^) and a ^3^MLCT in the monometallic references. Additionally, in this hypothesis, Coulombic effects should not be totally discarded, or, in other words, dual contributions from both localized–delocalized and proximal‐distal pictures could be at stake. A detailed orbital description of HE‐^3^MLCT from computation of its potential‐energy surface minimum requires more sophisticated theoretical methods, which will be studied in the future. Beyond the specific electronic configuration of HE‐^3^MLCT, a relatively mild electronic redistribution upon intra‐ligand ILET seems to be a key factor. This results in nested HE‐ and LE‐^3^MLCT potential energy surfaces and, in consequence, in a poor overlap of their vibrational wavefunctions. From a Fermi golden rule perspective, this affords poor Franck–Condon factors and a slow rate constant for IC via ILET.

Additionally, it is useful to estimate the Marcus reorganization energy λ according to

(1) $$\Delta G^{‡}=\frac{{\left(\lambda +\Delta G^{0}\right)}^{2}}{4\lambda }$$

From electrochemistry, Δ*G* ^0^ = −200 meV, and from our previous work,^[^ ^4^ ^]^ Δ*G* ^‡^ = 255 meV. Solving the quadratic equation gives two possible solutions for λ: 1363 and 29 meV. While the first one seems excessively large for an intra‐ligand ILET, λ = 29 meV seems reasonable. This very small reorganization energy pushes ILET into the inverted region where Δ*G* ^0^ < –λ, in line with the nested picture proposed above. This is probably one of the reasons for the slow ILET in the bimetallic complexes studied herein in comparison with {Ru(dppz)} compounds, where the exposed aromatic N atoms strongly interact with the solvent, leading to larger reorganizations and pushing ILET closer to the barrierless or normal Marcus regions.

Complementary fsTAS measurements were performed to shine light on the faster excited‐state dynamics in acetonitrile at room temperature, using 460 nm excitation (Figures S15–S17). Ru‐^dMe^bpy, Ru‐^5,5′dMe^bpy, and Ru‐^dMeO^bpy showed, next to the nanosecond‐lived HE‐^3^MLCT(L_m_^loc^) and LE‐^3^MLCT(L_m_^deloc^), an additional state, a vibrationally hot HE’‐^3^MLCT(bpy), living 23–40 ps (see Supporting Information for details). This state showcased a photoinduced absorption at 373, 375 and 378 nm for Ru‐^dMe^bpy, Ru‐^5,5′dMe^bpy, and Ru‐^dMeO^bpy, respectively, consistent with an MLCT state centered on the substituted ancillary bpy ligands. The short lifetimes indicated a fast bpy→L_m_ IC in the form of an inter‐ligand ILET from the ancillary ligand‐centered to the bridge‐centered states (Equation 3 and 3′). Finally, fsTAS of Ru‐^dCF3^bpy revealed, next to the nanosecond‐lived LE‐^3^MLCT(^dCF3^bpy), two additional excited states (Figure S18, see Supporting Information for details). On the one hand, the vibrationally hot state, hot LE’‐^3^MLCT(^dCF3^bpy), which relaxed to the long‐lived state in 78 ps. On the other hand, an 800 fs‐lived state showcasing photoinduced absorptions around 400 nm and at wavelengths > 500 nm, indicating a bridging‐ligand centered MLCT. Implicit was a fast dissipative L_m_→^dCF3^bpy ILET from the bridge‐centered to the ancillary ligand‐centered states (Equations 8 and 9).

**Figure 6:**
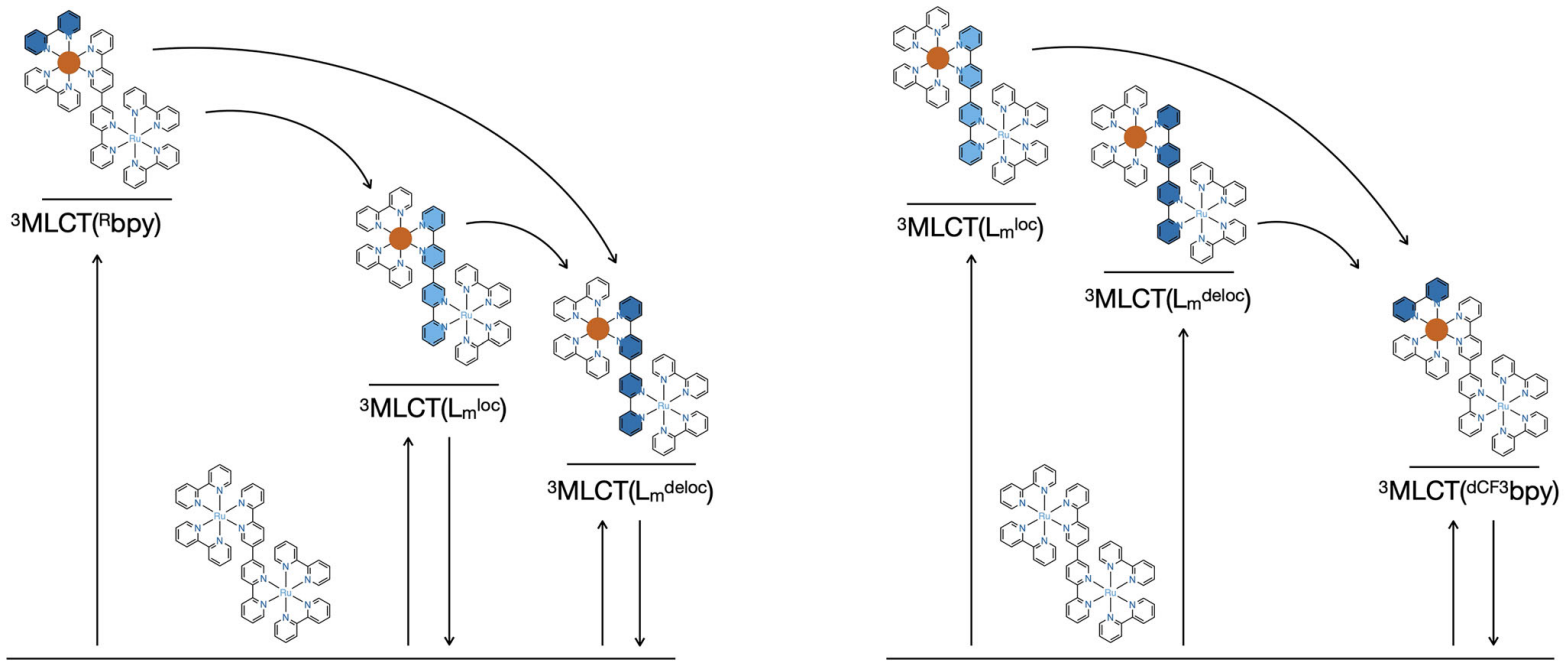
Schematic deactivation pathways for Ru‐bpy, Ru‐^dMe^bpy, Ru‐^5,5‐dMe^bpy, Ru‐^dMeO^bpy (left) and Ru‐^dCF3^bpy (right). Orange circle represents the oxidized metal center while the blue color represents the location of the electron following MLCT transition.

With all this information at hand, the deactivation pathways for Ru‐^dMe^bpy, Ru‐^5,5′dMe^bpy, and Ru‐^dMeO^bpy can be summarized by the following equations, and is depicted in Figure 6 (left):

(2) $$\begin{array}{ccc} & & Ru^{\mathrm{II}}{\left(\mathrm{bpy}\right)}_{2}\left(L_{m}\right)Ru^{\mathrm{II}}{\left(\mathrm{bpy}\right)}_{2}\overset{h\nu }{\to }\;Ru^{\mathrm{III}}{\left(\mathrm{bpy}\right)}_{2}\left(L_{m}^{\mathrm{deloc}•-}\right)Ru^{\mathrm{II}}{\left(\mathrm{bpy}\right)}_{2}\\ & & Ru^{\mathrm{II}}{\left(\mathrm{bpy}\right)}_{2}\left(L_{m}\right)Ru^{\mathrm{II}}{\left(\mathrm{bpy}\right)}_{2}\overset{h\nu }{\to }\;Ru^{\mathrm{III}}{\left(\mathrm{bpy}\right)}_{2}\left(L_{m}^{\mathrm{loc}•-}\right)Ru^{\mathrm{II}}{\left(\mathrm{bpy}\right)}_{2}\\ & & Ru^{\mathrm{II}}{\left(\mathrm{bpy}\right)}_{2}\left(L_{m}\right)Ru^{\mathrm{II}}{\left(\mathrm{bpy}\right)}_{2}\overset{h\nu }{\to }\;Ru^{\mathrm{III}}\left(\mathrm{bp}y^{•-}\right)\left(\mathrm{bpy}\right)\left(L_{m}\right)Ru^{\mathrm{II}}{\left(\mathrm{bpy}\right)}_{2} \end{array}$$

(3) $$\begin{array}{ccc} & & Ru^{\mathrm{III}}\left(\mathrm{bp}y^{•-}\right)\left(\mathrm{bpy}\right)\left(L_{m}\right)Ru^{\mathrm{II}}{\left(\mathrm{bpy}\right)}_{2}\;\overset{\mathrm{IC}/\mathrm{VR}}{\to }\;Ru^{\mathrm{III}}{\left(\mathrm{bpy}\right)}_{2}\left(L_{m}^{\mathrm{deloc}•-}\right)Ru^{\mathrm{II}}{\left(\mathrm{bpy}\right)}_{2}\\ & & Ru^{\mathrm{III}}\left(\mathrm{bp}y^{•-}\right)\left(\mathrm{bpy}\right)\left(L_{m}\right)Ru^{\mathrm{II}}{\left(\mathrm{bpy}\right)}_{2}\overset{\mathrm{IC}/\mathrm{VR}}{\to }\;Ru^{\mathrm{III}}{\left(\mathrm{bpy}\right)}_{2}\left(L_{m}^{\mathrm{loc}•-}\right)Ru^{\mathrm{II}}{\left(\mathrm{bpy}\right)}_{2} \end{array}$$

(4) $$Ru^{\mathrm{III}}{\left(\mathrm{bpy}\right)}_{2}\left(L_{m}^{\mathrm{loc}•-}\right)\left(L_{m}\right)Ru^{\mathrm{II}}{\left(\mathrm{bpy}\right)}_{2}\;\overset{\mathrm{IC}/\mathrm{VR}}{\to }\;Ru^{\mathrm{III}}{\left(\mathrm{bpy}\right)}_{2}\left(L_{m}^{\mathrm{deloc}•-}\right)Ru^{\mathrm{II}}{\left(\mathrm{bpy}\right)}_{2}$$

(5) $$Ru^{\mathrm{III}}{\left(\mathrm{bpy}\right)}_{2}\left(L_{m}^{\mathrm{loc}•-}\right)\left(L_{m}\right)Ru^{\mathrm{II}}{\left(\mathrm{bpy}\right)}_{2}\;\overset{k_{r},k_{\mathrm{nr}}}{\to }\;Ru^{\mathrm{II}}{\left(\mathrm{bpy}\right)}_{2}\left(L_{m}\right)Ru^{\mathrm{II}}{\left(\mathrm{bpy}\right)}_{2}$$

(6) $$Ru^{\mathrm{III}}{\left(\mathrm{bpy}\right)}_{2}\left(L_{m}^{\mathrm{deloc}•-}\right)\left(L_{m}\right)Ru^{\mathrm{II}}{\left(\mathrm{bpy}\right)}_{2}\;\overset{k_{r},k_{\mathrm{nr}}}{\to }\;Ru^{\mathrm{II}}{\left(\mathrm{bpy}\right)}_{2}\left(L_{m}\right)Ru^{\mathrm{II}}{\left(\mathrm{bpy}\right)}_{2}$$

In contrast, for Ru‐^dCF3^bpy, the deactivation pathways are described by the following equations, and depicted in Figure 6 (right):

(7)

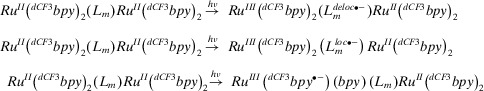



(8)

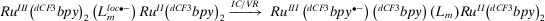



(9)

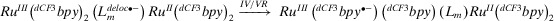



(10)

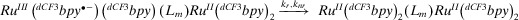




A survey of literature reports on ILET in Ru(II), Os(II), Ir(III), and Re(I) complexes affords a rather diverse panorama, with only few examples of slow ILET in the nanosecond timescale including both intra‐ligand and inter‐ligand ILET.^[^ ^2^ ^]^ For example, among a fair amount of Ru(II) and Os(II) compounds bearing dppz‐type ligands, only [Os(TAP)_2_(dppp2)]^2+^ (where TAP is 1,4,5,8‐tetraazaphenanthrene and dppp2 is pyrido[2′,3′:5,6]pyrazino[2,3‐f][1,10]phenanthroline) showed multi‐exponential emission decay,^[^ ^23^ ^]^ perhaps due to slow ILET, while the other members of the family undergo ILET in the picosecond timescale.^[^ ^19^, ^20^, ^21^, ^24^, ^25^, ^26^, ^27^, ^28^, ^29^, ^30^, ^31^, ^32^, ^33^, ^34^, ^35^, ^36^, ^37^, ^38^, ^39^, ^40^ ^]^ Among alkynyl‐substituted phen or bpy Ru(II) complexes, unsymmetrically 4‐substituted phen systems afforded dual emission with nanosecond lifetimes,^[^ ^41^, ^42^ ^]^ implying slow ILET, but symmetric 4,7‐disubstitution or unsymmetric 3‐ or 5‐substitution accelerated ILET to the picosecond timescale.^[^ ^41^, ^42^, ^43^, ^44^, ^45^, ^46^, ^47^ ^]^ Globally, it appears that slight structural or electronic changes have drastic consequences on ILET rate constants. For example, single emissions were reported for monometallic [Ru(bpy)_2_(L_m_)]^2+^, [Ru(deeb)_2_(L_m_)]^2+^ (where deeb is 4,4′‐diethylester‐2,2′‐bipyiridine) and their bimetallic analogues bearing {Pt^II^Cl_2_} and {Re^I^(CO)_3_Cl},^[^ ^48^, ^49^ ^]^ but dual emissions were observed for the bimetallic analogues containing {Pd^II^Cl_2_}^[^ ^48^ ^]^ and for Ru‐bpy, Ru‐^dMe^bpy, Ru‐^5,5′dMe^bpy, and Ru‐^dMeO^bpy studied herein. Rigorous systematic studies using varied (time‐resolved) spectroscopic and electrochemical techniques, which clearly reveal structure‐property correlations leading to nanosecond ILET, seem to be scarce in the literature. It is in this context that the results informed here stand out, where an arsenal of different techniques allowed to provide useful design guidelines for anti‐dissipative chromophores. Against our initial hypothesis, inter‐ligand ILET was much faster than intra‐ligand ILET for this series of quaterpyridine‐bridged Ru polypyridines. Synthetic efforts should be devoted to chromophoric ligands with poor intra‐ligand electronic coupling, like in the *meta*‐connected quaterpyridine explored here, in clear contrast to *para*‐connected analogues where intra‐ligand ILET is accelerated.^[^ ^4^ ^]^ Rather simple, non‐chromophoric co‐ligands could be used, since they would not contribute to anti‐dissipative activity. However, the use of ancillary ligands substituted with strong electron‐ accepting groups is detrimental, since it favors a fast depopulation of the anti‐dissipative ligand via inter‐ligand ILET.

### Excited‐State Reactivity

**Figure 7:**
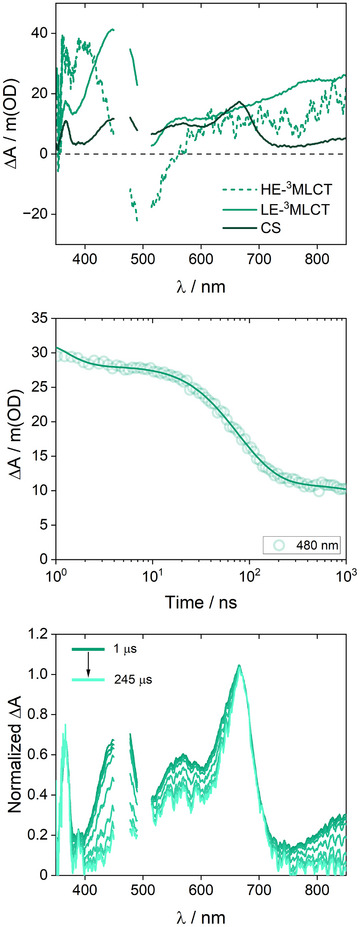
Top: Species associated differential spectra extracted upon nsTAS (time delays smaller than 1 µs) of 0.1 mM Ru‐^dMeO^bpy in the presence of 8 mM TTA in acetonitrile at room temperature. Middle: Kinetic trace at 480 nm (dots) and fit (lines). Bottom: Spectral reshaping for time delays greater than 1 µs. Differential spectra at selected time delays are shown, normalized to the maximum assigned to TTA^•+^.

As in Ru‐bpy, HE‐^3^MLCT(L_m_^loc^) in Ru‐^dMe^bpy, Ru‐^5,5‐dMe^bpy, and Ru‐^dMeO^bpy should be sufficiently long‐lived to undergo bimolecular electron transfer and could hence be intercepted before IC for anti‐dissipative energy conversion.^[^ ^4^ ^]^ Ru‐^dMeO^bpy, having the longest HE‐^3^MLCT(L_m_^loc^) excited‐state lifetime with 44 ns, was chosen for the proof‐of‐concept. An 8 mM solution of tri‐tolylamine (TTA, 0.93 V versus NHE) as an electron donor was used, serving as model for anti‐dissipative catalyst activation. This reaction is similar to catalyst activation by (sacrificial) electron donors in reductive photocatalysis. It was investigated by means of nsTAS in the time range 1 ns – 400  µs. Formation of charge‐separated (CS) states was clearly observed in the appearance of intense photoinduced absorptions between 400 and 850 nm ascribed to the reduced species, and a sharp absorption maximum at 670 nm corresponding to the oxidized TTA (TTA^•+^) (Figure 7).^[^ ^50^, ^51^ ^]^ CS species were essentially completely formed at a time delay of 200 ns upon quenching of HE‐^3^MLCT(L_m_^loc^) and LE‐^3^MLCT (L_m_^deloc^), as shown in the kinetic trace at 480 nm (Figure 7). Species‐associated spectra of both excited states and of the CS product mixture were recovered using a target analysis over the first microsecond after the laser pulse. Both excited states showed decreased lifetimes (15 and 70 ns respectively) relative to the experiment in the absence of TTA, confirming that both excited states react independently with the electron donor (Equations 11 and 12). In fact, quenching constants of 2.1 x 10^9^ and 5.5 x 10^8^ M^−1^cm^−1^ were derived for HE‐^3^MLCT(L_m_^loc^) and LE‐^3^MLCT (L_m_^deloc^), respectively (Figure S20), which are close to the diffusion limit. Next, the nature of the formed CS products was investigated. Like for Ru‐bpy, different CS species, both including TTA^•+^ (sharp signal at 670 nm) but different radical anions, were produced in the photoinduced electron transfer from TTA to coexisting HE‐^3^MLCT(L_m_^loc^) and LE‐^3^MLCT (L_m_^deloc^). This was evident in the significant spectral reshaping that accompanied a general intensity loss in the microsecond timescale (Figure 7). Photoinduced absorptions above 700 nm and below 570 nm decayed faster than the sharp absorption at 670 nm ascribed to TTA^•+^. This is consistent with the differential absorption features of the two‐electron reduced species evolving faster than those of the one‐electron reduced species. Therefore, we assigned the observed microsecond dynamics as the decay of L_m_^loc•–^ and L_m_^deloc•–^ (Equations 13 and 14), i.e., recombination of the respective radical anions with TTA^•+^, taking place in a few microseconds and in hundreds of microseconds, respectively. No evidence of L_m_^loc•–^‐to‐L_m_^deloc•–^ ILET / IC (Equation 15) was observed. However, this process might be difficult to resolve from a L_m_^loc•–^ decay to the ground state, since spectroelectrochemistry suggests that the spectral features of the former are probably more intense than those from the latter, so it cannot be ruled out at this stage.

(11) $$Ru^{\mathrm{III}}{\left(\mathrm{bpy}\right)}_{2}\left(L_{m}^{\mathrm{loc}•-}\right)Ru^{\mathrm{II}}{\left(\mathrm{bpy}\right)}_{2}+\mathrm{TTA}\to \;Ru^{\mathrm{II}}{\left(\mathrm{bpy}\right)}_{2}\left(L_{m}^{\mathrm{loc}•-}\right)Ru^{\mathrm{II}}{\left(\mathrm{bpy}\right)}_{2}+\mathrm{TT}A^{•+}$$

(12) $$Ru^{\mathrm{III}}{\left(\mathrm{bpy}\right)}_{2}\left(L_{m}^{\mathrm{deloc}•-}\right)Ru^{\mathrm{II}}{\left(\mathrm{bpy}\right)}_{2}+\mathrm{TTA}\to \;Ru^{\mathrm{II}}{\left(\mathrm{bpy}\right)}_{2}\left(L_{m}^{\mathrm{deloc}•-}\right)Ru^{\mathrm{II}}{\left(\mathrm{bpy}\right)}_{2}+\mathrm{TT}A^{•+}$$

(13) $$Ru^{\mathrm{II}}{\left(\mathrm{bpy}\right)}_{2}\left(L_{m}^{\mathrm{loc}•-}\right)Ru^{\mathrm{II}}{\left(\mathrm{bpy}\right)}_{2}+\mathrm{TT}A^{•+}\to Ru^{\mathrm{II}}{\left(\mathrm{bpy}\right)}_{2}\left(L_{m}\right)Ru^{\mathrm{II}}{\left(\mathrm{bpy}\right)}_{2}+\mathrm{TTA}$$

(14) $$Ru^{\mathrm{II}}{\left(\mathrm{bpy}\right)}_{2}\left(L_{m}^{\mathrm{deloc}•-}\right)Ru^{\mathrm{II}}{\left(\mathrm{bpy}\right)}_{2}+\mathrm{TT}A^{•+}\to Ru^{\mathrm{II}}{\left(\mathrm{bpy}\right)}_{2}\left(L_{m}\right)Ru^{\mathrm{II}}{\left(\mathrm{bpy}\right)}_{2}+\mathrm{TTA}$$

(15) $$Ru^{\mathrm{II}}{\left(\mathrm{bpy}\right)}_{2}\left(L_{m}^{\mathrm{deloc}•-}\right)Ru^{\mathrm{II}}{\left(\mathrm{bpy}\right)}_{2}\to Ru^{\mathrm{II}}{\left(\mathrm{bpy}\right)}_{2}\left(L_{m}^{\mathrm{loc}•-}\right)Ru^{\mathrm{II}}{\left(\mathrm{bpy}\right)}_{2}$$

Notably, application of the usual equation to calculate excited‐state reduction potentials (Equation 16) is not straightforward when the reduction products of LE‐^3^MLCT (L_m_^deloc^) and HE‐^3^MLCT (L_m_^loc^) are different species (Equations 11 and 12). This would require different values of *E*_red_ for each excited state.

(16)

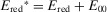




**Figure 8:**
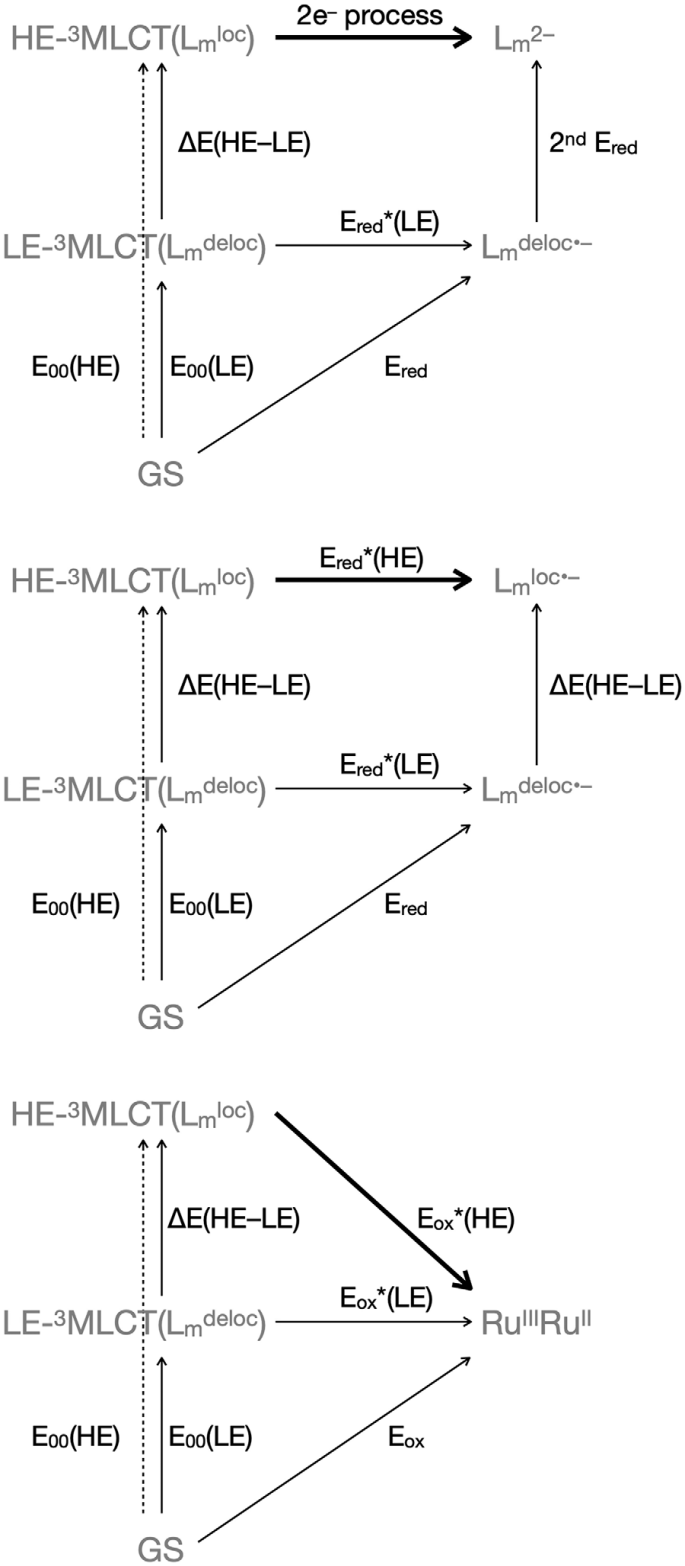
Thermodynamic cycles for a two‐electron reduction (top), a one‐electron reduction (middle) and a one‐electron oxidation of HE‐^3^MLCT(L_m_^loc^), highlighted as bold arrows.

For LE‐^3^MLCT(L_m_^deloc^), consideration of the first reduction potential offers a good approximation of *E*_red_*(LE) (Table 2). In contrast, for HE‐^3^MLCT(L_m_^loc^), a perhaps intuitive approximation of *E*_red_*(HE) that utilizes the second reduction potential is flawed. On the one hand, this potential value includes a coulombic penalty that originates from the implicit first reduction event, leading to the presence of two electrons in the two‐electron reduced species, as determined by electrochemistry. On the other hand, the second *E*_red_ involves the generation of a two‐electron reduced species, whose production from HE‐^3^MLCT(L_m_^loc^) requires a two‐electron process (Figure 8). Instead, the desired *E*_red_*(HE) involves a one‐electron reduction of HE‐^3^MLCT(L_m_^loc^) (Figure 8, middle). To circumvent this, one can simply assume that the energy difference between L_m_^loc•–^ and L_m_^deloc•–^ is approximately the spectroscopically derived *ΔE*(HE–LE). Under this assumption, *E*_red_* for LE‐^3^MLCT(L_m_^deloc^) and HE‐^3^MLCT(L_m_^loc^) are equal (Figure 8). This is a consequence of the fact that both the reactant and product of excited‐state reduction are high‐energy species (Equation 11). The rate constants for photoinduced electron transfer derived here for Ru‐^dMeO^bpy, 2.1 x 10^9^ and 5.5 x 10^8^ M^−1^cm^−1^, respectively for HE‐^3^MLCT(L_m_^loc^) and LE‐^3^MLCT (L_m_^deloc^), are smaller than those previously obtained for Ru‐bpy, of 7.9 x 10^9^ and 4.0 x 10^9^ M^−1^cm^−1^, in line with a 150 meV smaller *E*_00_, and therefore *E*_red_*, for Ru‐^dMeO^bpy. Bimolecular quenching constants for HE‐^3^MLCT(L_m_^loc^) with TTA are larger by a factor of 2–4 relative to those for LE‐^3^MLCT (L_m_^deloc^). Photoreduction of HE‐^3^MLCT(L_m_^loc^) directly affording L_m_^deloc•–^, which is not considered in our simplified scheme, might be the reason behind this (Figure 8).

The reactivity of HE‐^3^MLCT(L_m_^loc^) in Ru‐^dMeO^bpy with the model electron acceptor 1,4‐benzoquinone (BzQ, −0.165 V versus NHE)^[^ ^52^ ^]^ was also explored. Shortened lifetimes for HE‐^3^MLCT(L_m_^loc^) and LE‐^3^MLCT(L_m_^deloc^) were observed, but, as in similar systems,^[^ ^53^ ^]^ the lifetimes of the CS species are shorter than their generation, impeding the accumulation of detectable populations. The case for excited‐state oxidation potentials is however different from that of excited‐state reduction potentials. One‐electron oxidation of both LE‐^3^MLCT(L_m_^deloc^) and HE‐^3^MLCT(L_m_^loc^) presumably generate the same product (Equations 17 and 18), so the same *E*_ox_ can be used for both excited states in Equation 19. Therefore, the driving force for HE‐^3^MLCT(L_m_^loc^) directly benefits from *ΔE*(HE–LE), which enters in Equation 19 through the corresponding *E*_00_(HE). This is illustrated in Figure 8, bottom. For Ru‐^dMeO^bpy, *ΔE*(HE–LE) is only 50 meV (Table 2), so, in this case, only slight differences are expected in excited‐state oxidation reactivity. In fact, Stern–Volmer experiments resulted in very similar bimolecular quenching rate constants of 6.9 x 10^9^ and 5.9 x 10^9^ M^−1^cm^−1^, respectively for HE‐^3^MLCT(L_m_^loc^) and LE‐^3^MLCT (L_m_^deloc^) (Figure S20).

(17) $$Ru^{\mathrm{III}}{\left(\mathrm{bpy}\right)}_{2}\left(L_{m}^{\mathrm{loc}•-}\right)Ru^{\mathrm{II}}{\left(\mathrm{bpy}\right)}_{2}+\mathrm{BzQ}\to \;Ru^{\mathrm{III}}{\left(\mathrm{bpy}\right)}_{2}\left(L_{m}\right)Ru^{\mathrm{II}}{\left(\mathrm{bpy}\right)}_{2}+\mathrm{Bz}Q^{•-}$$

(18) $$Ru^{\mathrm{III}}{\left(\mathrm{bpy}\right)}_{2}\left(L_{m}^{\mathrm{deloc}•-}\right)Ru^{\mathrm{II}}{\left(\mathrm{bpy}\right)}_{2}+\mathrm{BzQ}\to \;Ru^{\mathrm{III}}{\left(\mathrm{bpy}\right)}_{2}\left(L_{m}\right)Ru^{\mathrm{II}}{\left(\mathrm{bpy}\right)}_{2}+\mathrm{Bz}Q^{•-}$$

(19)

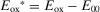




This analysis highlights the versatility of anti‐dissipative chromophores, which can be used as photo‐oxidants to produce HE anions which preserve the spared energy and can, in a later step, engage in energy‐demanding reactivity. Alternatively, they can be utilized as photo‐reductants to directly exploit the non‐dissipated energy.

## Conclusion

HE excited states were precisely identified on ancillary bpy ligands and on a localized portion of the bridge, probably distal to the transiently oxidized Ru(III), in a series of quaterpyridine‐bridged ruthenium polypyridines. This level of detail was mainly enabled thanks to the radical anion photoinduced absorptions of each excited state, observed between 360 and 460 nm using nsTAS and fsTAS, in combination with electrochemistry and spectroelectrochemistry.

This study provides design guidelines for anti‐dissipative chromophores. Substitution of the ancillary ligands is, in addition to the poor electronic coupling brought by the *meta* topology of the bridge, a key parameter to control dissipative IC/VR pathways. The addition of electron accepting groups on the ancillary ligands stabilized their ^3^MLCT making them the LE excited states, which resulted in contributions from bridging ligand‐centered HE‐^3^MLCT only in the hundreds‐of‐femtoseconds timescale with a negligible energy barrier for HE→LE IC. This is therefore not beneficial for anti‐dissipative energy conversion strategies and should be avoided. On the other hand, the addition of electron donating moieties on the ancillary ligands led to the destabilization of their ^3^MLCT states ligands above the bridging ligand‐centered LE‐^3^MLCT states. This scenario afforded IC in the form of a slow ILET within different portions of the bridging ligand. ILET rates and *ΔE*(HE–LE) are moderately influenced by ancillary ligand substitution. Lifetimes of 5–40 ns and HE–LE energy differences of 50–120 meV were obtained. While this is attractive to develop anti‐dissipative energy conversion, electron donating ligands slightly shrank *ΔE*(HE–LE). Therefore, utilization of very strong electron donating groups should be circumvented in the design. Remarkably, our fsTAS/nsTAS studies indicate that inter‐ligand IC processes in the form of ILET are much faster than intra‐ligand ILET. In this context, different substitution patterns on the bridge should be explored as a promising strategy to gain further control over anti‐dissipative behavior in these systems.

Finally, our analysis highlights the versatility of HE excited states in terms of their photo‐reduction and photo‐oxidation abilities, and the energetics that should be considered in the design of anti‐dissipative energy‐conversion schemes based upon them.

## Supporting Information

Experimental details, calculations, additional discussion of fitting models, nanosecond and femtosecond transient absorption spectroscopy.

## Conflict of Interests

The authors declare no conflict of interest.

## Supporting information



Supporting Information

## Data Availability

The data that support the findings of this study are available from the corresponding author upon reasonable request.
