# Supplementary material for: Nature of Anti‐Dissipative High‐Energy Excited States in Quaterpyridine‐Bridged Ruthenium Complexes
Source: Angew Chem Int Ed Engl. 2025 Jun 30;64(33):e202507738. doi: 10.1002/anie.202507738 (PMC12338390; doi:10.1002/anie.202507738)
Supplement: Supplementary file 1 — Supporting Information [file ANIE-64-e202507738-s001.pdf]

Supporting Information for

Nature of Anti-Dissipative High-Energy Excited States in Quaterpyridine-Bridged Ruthenium Complexes

Noémie Chantry,[a] Agustina Cotic,[b,c] Simon De Kreijger,[a] Riccardo Di Forti,[e,f] Benjamin Elias,[a] Ludovic Troian-Gautier,[a,d] * and Alejandro Cadranel [a,b,e,f] *

[a] Université catholique de Louvain (UCLouvain), Institut de la Matière Condensée et des Nanosciences (IMCN), Molecular Chemistry, Materials and Catalysis (MOST), Place Louis Pasteur 1, bte L4.01.02, 1348 Louvain-la-Neuve, Belgium

[b] Universidad de Buenos Aires, Facultad de Ciencias Exactas y Naturales, Departamento de Química Inorgánica, Analítica y Química Física, Pabellón 2, Ciudad Universitaria, C1428EHA, Buenos Aires, Argentina.

[c] CONICET − Universidad de Buenos Aires, Instituto de Química-Física de Materiales, Medio Ambiente y Energía (INQUIMAE), Pabellón 2, Ciudad Universitaria, C1428EHA, Buenos Aires, Argentina.

[d] Wel Research Institute, Avenue Pasteur 6, 1300 Wavre, Belgium

[e] Friedrich-Alexander-Universität Erlangen-Nürnberg (FAU), Physical Chemistry I, Egerlandstr. 3, 91058, Erlangen, Germany.

[f] Friedrich-Alexander-Universität Erlangen-Nürnberg (FAU), Interdisciplinary Center for Molecular Materials, Egerlandstr. 3, 91058, Erlangen, Germany.

*Correspondence to: Ludovic.Troian@uclouvain.be, ale.cadranel@fau.de

Experimental Details....S2
Syntheses....S5
NMR Spectroscopy....S7
Spectroelectrochemistry....S9
Steady-state Emission Spectroscopy....S10
Time-Resolved Emission Spectroscopy....S12
Nanosecond Transient Absorption Spectroscopy....S13
Femtosecond Transient Absorption Spectroscopy....S17
Stern-Volmer Studies....S21
References....S22

Experimental Details

Materials. Acetonitrile (VWR), butyronitrile (VWR), acetonitrile D3 99.8% (Eurisotop), ammonium hexafluorophosphate 99% (FluoroChem), anhydrous acetonitrile 99.8% (Sigma), cyclooctadiene 99+% (Thermo Scientific), dichlorobenzene (Acros), diethyl ether (VWR), ethanol (VWR), ethylene glycol (Acros), potassium nitrate 99+% (Acros), silver nitrate 99% (Acros Organics), tetra-n-butylammonium hexafluorophosphate 98+% (TCI) were purchased from commercial suppliers and used as received. Water was purified with a Millipore Milli-Q system. 4,4'-bis(trifluoromethyl)-2,2'-bipyridine, 4,4'-dimethyl-2,2'-bipyridine, 5,5'-dimethyl-2,2'-bipyridine, 4,4'-dimethoxy-2,2'-bipyridine, Ru-bpy, $[Ru(bpy)_3]^{2+}$, $[Ru(^{dMe}bpy)_3]^{2+}$, $[Ru(^{5,5'dMe}bpy)_3]^{2+}$, $[Ru(^{dMeO}bpy)_3]^{2+}$, $[Ru(^{dCF3}bpy)_3]^{2+}$, tri-*p*-tolylamine and 1,4-benzoquinone were already available in the lab.

Microwave synthesis. Microwave (MW) syntheses were performed on a Milestone MicroSYNTH labstation under magnetic stirring. Typically, the conditions allowed the vessels to reach the desired temperature in 5 minutes and the vessels were held at the desired temperature for the indicated period of time.

NMR. Characteristic NMR spectra were obtained at room temperature using a Bruker AC-300 Avance II (300 MHz) or Bruker AM-500 (500 MHz) at 20 °C. Solvent residual peaks were used as internal standards for ^1H (δ = 1.94 ppm for CD_3CN) chemical shift referencing. NMR spectra were processed using MNOVA.

Mass Spectrometry. High-resolution ESI-MS was performed with a Q-Extractive orbitrap from ThermoFisher using reserpine as the internal standard. Samples were ionized by electrospray ionization (ESI).

Electrochemistry. Differential pulse voltammetry was performed with an Autolab PGSTAT 100 potentiostat using a standard three-electrode cell, i. e., a glassy carbon disk working electrode (approximate area = 3 mm^2), a platinum wire counter electrode, and an aqueous Ag/AgCl reference electrode (salt bridge: 3 M KCl/saturated AgCl). Experiments were performed in dry acetonitrile with 0.1 M $TBAPF_6$ as electrolyte at a scan rate of 0.1 to 0.3 Vs^{-1}. The sample, with a

complex concentration of 1 mM, was purged with argon before the measurement. For comparison purposes, the electrochemical potentials were converted to SCE by subtracting 0.036 V.

Spectroelectrochemistry. Spectroelectrochemistry was measured in an OTTLE (Optically Transparent Thin-Layer Electrochemical) cell[1] (acquired from the University of Reading, UK) consisting of CaF_2 windows, a polymeric spacer with a thickness of around 200 μm, and an electrode set involving a Pt mesh as working electrode, another Pt mesh as counter electrode and a Ag sheet as a pseudo-reference electrode.

Steady-state absorption. Steady-state absorption was performed in quartz cuvettes with 1 cm path length, using a Shimadzu UV-1900i UV-Vis spectrophotometer double beam spectrometer (190 to 1100 nm) or in a Agilent Cary 60.

Steady-state emission/excitation. Steady-state emission/excitation spectroscopy was recorded on an FS5 spectrofluorometer from Edinburgh Instruments or with a Varian Cary Eclipse using argon-saturated solutions in 1 cm optical path cuvettes. Spectra were corrected using the response function provided by the manufacturer. For 80K measurements, an Optistat DN cryostat from Oxford Instruments was employed, and Franck-Condon lineshape analysis was performed using a reported software.[2] Photoluminescence quantum yields were obtained by comparative actinometry using $[Ru(bpy)_3]^{2+}$ as a reference.[3]

Time-Resolved Emission. Emission lifetimes were measured using a Fluorolog 3 time-correlated photon counting (TCSPC) instrument from Horiba Jovin Yvon, a SuperK Fianium FIU6PP supercontinuum laser from NKT Photonics as the excitation source, and a R3809U-50 MCP photomultiplier from Hammamatsu. Alternatively, an FS5 Spectrofluorometer from Edinburgh Instruments equipped with a time-correlated single photon counting module was utilized, together with E 450$\pm$5 nm diode laser (EPL-450, 85 ps pulse width at 10 MHz) for excitation.

Transient Absorption. Transient absorption (TA) experiments were conducted using an Astrella-F-1K amplified Ti:sapphire femtosecond laser system from Coherent, operating at a repetition rate 1kHz, 5.5 W power (5 mJ pulse energy), with a pulse duration of 80 fs. TA pump/probe Helios (fsTA) and EOS (nsTA) detection systems from Ultrafast Systems were utilized. In the fsTA experiment, an optical delay line placed in the probe beam pathway allowed for time delays up to 7.2 ns. White light was generated focusing a fraction of the fundamental 800 nm

output onto a 2 mm (vis) sapphire crystal. In the nsTA experiments, the white light (~370 to >1600 nm) source was a built-in photonic crystal fiber supercontinuum pulsed laser source with a fundamental of 1064 nm at 2 kHz output frequency and pulse width of approximately 1 ns. A 1.2 mJ fraction of the fundamental is used for pump beam generation by a TOPAS Prime from Light Conversion with standard NirUVis extension. Pump energy was typically 1000 nJ. A depolarizer was placed in the pump beam to avoid rotational dynamics. Bandpass filters with ± 5 or ± 10 nm were used to ensure low spectral width and to exclude 800 nm photons. Excitation spot diameters were typically 150-200 μm (1/e) (affording pump fluences around 5-10 mJ/cm^2) and were ensured to be larger than those of the probe beam. All measurements were conducted in a 2 mm quartz cuvette under argon atmosphere, using solutions with room temperature absorbances of 0.5-0.7. To analyze transient absorption data, we used a suggested procedure.[4] We start with SVD and global analysis, using an all-sequential decay model that provides evolution associated spectra of potentially intervening species, to determine the number of decaying species that participate in the decay cascade. However, this doesn't necessary yield differential spectra with genuine physicochemical meaning. Afterwards, a target analysis is applied, using specific target models that result in species associated spectra with true physicochemical meaning. Obtained data were treated by SVD, global and target analyses using the R- package TIMP and GloTarAn.[4–6] The instrument response function (IRF) and dispersion (chirp of the white light pulse) were modelled and taken into account during the fitting procedure.

Smoothed HE-^{3}MLCT spectra were obtained using a Savitzky-Golay method implemented in Origin 2024, with a 2nd order polynomial, a window of 80 points and no boundary conditions.

Syntheses

Quaterpyridine Lm ligand was synthesized as previously reported. [7] Complexes were synthesized using the following procedure:

Ru(COD). Ru(COD) was synthesized following a modified procedure from that one published.[8] 3 g of $RuCl_3.3H_2O$ was dissolved in degassed ethanol in a three-neck round-bottom flask. An excess of cyclooctadiene (8.2 equivalents) was subsequently added. The mixture was heated under reflux for 5 hours. After cooling to room temperature, the resulting precipitate was washed three times with 35 mL of ether, yielding a brown powder (2.874 g, 89%).

$[Ru(LL)_2Cl_2]$. Metal precursors were obtained from a modified procedure.[9] LL stands for 4,4'-dimethyl-2,2'-bipyridine, 5,5'-dimethyl-2,2'-bipyridine, 4,4'-dimethoxy-2,2'-bipyridine or 4,4'-bis(trifluoromethyl)-2,2'-bipyridine. Ru(COD) and LL (2.2 eq) were dissolved in 6 mL dichlorobenzene. The solution was then purged with argon for 15 minutes and heated at 150°C for 2 hours. After cooling to room temperature, 40 mL of ether was added to promote precipitation. The resulting precipitate was collected by filtration and washed with ether. The obtained product was used directly in the next step without further purification.

$[Ru(LL)_2(L_m)Ru(LL)_2]^{4+}$. The complexation of all the compounds was carried out using a slightly modified procedure from that one reported.[7] L_m ligand (0.025 mg, 0.0806 mmol) and $[Ru(LL)_2Cl_2]$ (2.6 eq.) were transferred un in a microwave tube with a large excess of $AgNO_3$ (0.1368 g, 0.8055 mmol, 10 eq.). A 9:1 ethylene glycol/water (27 mL) mixture was then added and subsequently purged with argon for 15 minutes. The reaction mixture was heated at 180°C for 1 hour in a microwave reactor. After reaction completion, saturated ammonium hexafluorophosphate solution was gradually added under stirring, which induced the precipitation. The resulting precipitate was collected by filtration and washed thoroughly with water. The solid was then purified by flash chromatography on SiO_2 (acetonitrile/saturated aqueous KNO_3/water: v/v/v: 7/1/0.5). The desired fractions were collected and evaporated under reduced pressure. When necessary, a complementary LH20 size exclusion chromatography was conducted in acetonitrile. The resulting products were isolated as the hexafluorophosphate salts after ion metathesis. The

powder was finally dissolved in acetonitrile, filtered through a 0.45 µm PTFE syringe filter and the solvent was removed under reduced pressure to yield the pure complexes.

Ru-^{dMe}bpy

Red powder (0.073 g – 38%) - ^{1}H NMR (300 MHz, CD_3CN) δ 8.49 (td, *J* = 8.5, 7.1, 3.5 Hz, 6H), 8.39 – 8.27 (m, 14H), 8.04 (tt, *J* = 7.9, 1.8 Hz, 4H), 7.90 (ddd, *J* = 8.5, 5.0, 2.0 Hz, 4H), 7.69 (t, *J* = 4.2 Hz, 4H), 7.58 (d, *J* = 5.8 Hz, 2H), 7.51 (dd, *J* = 8.9, 5.8 Hz, 4H), 7.48 – 7.35 (m, 12H), 7.27 – 7.13 (m, 12H). HRMS (ESI): m/z calculated for $[C_{68}H_{62}N_{12}Ru_2]^{4+}$, 312.5821; found: 312.5829.

Ru-$^{5,5'dMe}$bpy

Red powder (0.097 g – 51%) - ^{1}H NMR (300 MHz, CD_3CN) δ 8.57 – 8.46 (m, 6H), 8.37 – 8.20 (m, 14H), 8.14 – 7.99 (m, 4H), 7.89 – 7.81 (m, 16H), 7.79 – 7.73 (m, 2H), 7.69 (td, *J* = 5.0, 4.5, 2.3 Hz, 4H), 7.49 (ddd, *J* = 7.0, 4.0, 1.6 Hz, 4H), 7.45 – 7.38 (m, 10H), 7.37 – 7.31 (m, 2H). HRMS (ESI): m/z calculated for $[C_{68}H_{62}N_{12}Ru_2]^{4+}$, 313.0824; found: 313.0829.

Ru-^{dMeO}bpy

Red powder (0.017 g – 8%) - *^{1}H NMR (300 MHz, CD3CN) δ 8.52 – 8.42 (m, 6H), 8.04 – 7.97 (m, 10H), 7.94 (d, J = 2.4 Hz, 6H), 7.90 – 7.82 (m, 4H), 7.77 (t, J = 5.5 Hz, 4H), 7.51 – 7.32 (m, 18H), 7.01 – 6.89 (m, 10H), 6.85 (dt, J = 6.4, 3.0 Hz, 4H).* HRMS (ESI): m/z calculated for $[C_{68}H_{62}N_{12}O_8Ru_2]^{4+}$, 344.5728; found: 344.5728.

Ru-^{dCF3}bpy

Red powder (0.094 g – 40%) - ^{1}H NMR (300 MHz, CD_3CN) δ 8.97 – 8.85 (m, 8H), 8.56 (d, *J* = 8.2 Hz, 2H), 8.49 (d, *J* = 8.6 Hz, 2H), 8.18 – 8.06 (m, 4H), 8.05 – 7.97 (m, 4H), 7.95 – 7.81 (m, 6H), 7.68 (m, 10H), 7.45 (ddd, *J* = 7.5, 5.8, 1.3 Hz, 2H). HRMS (ESI): m/z calculated for $[C_{68}H_{38}F_{24}N_{12}Ru_2]^{4+}$, 421.0259; found: 421.0265.

NMR Spectroscopy

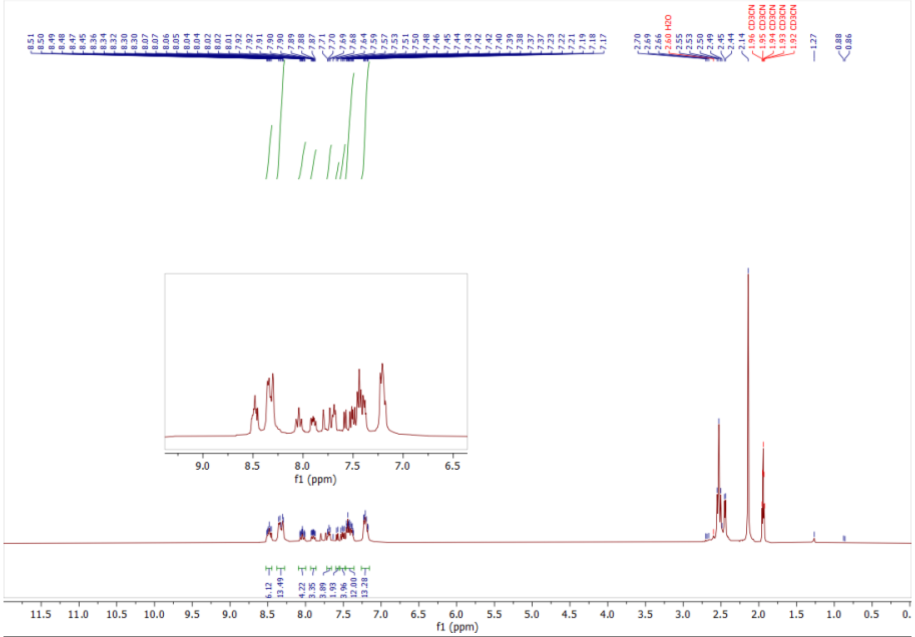

Figure S1. ^{1}H-NMR spectrum of Ru-^{dMe}bpy in CD_3CN.

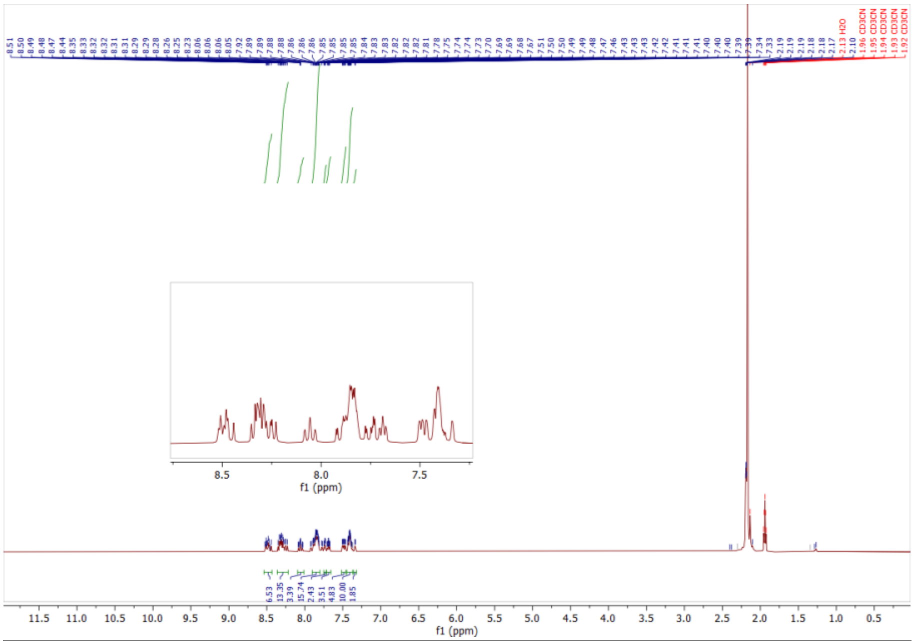

Figure S2. ^{1}H-NMR spectrum of Ru-$^{5,5'dMe}$bpy in CD_3CN.

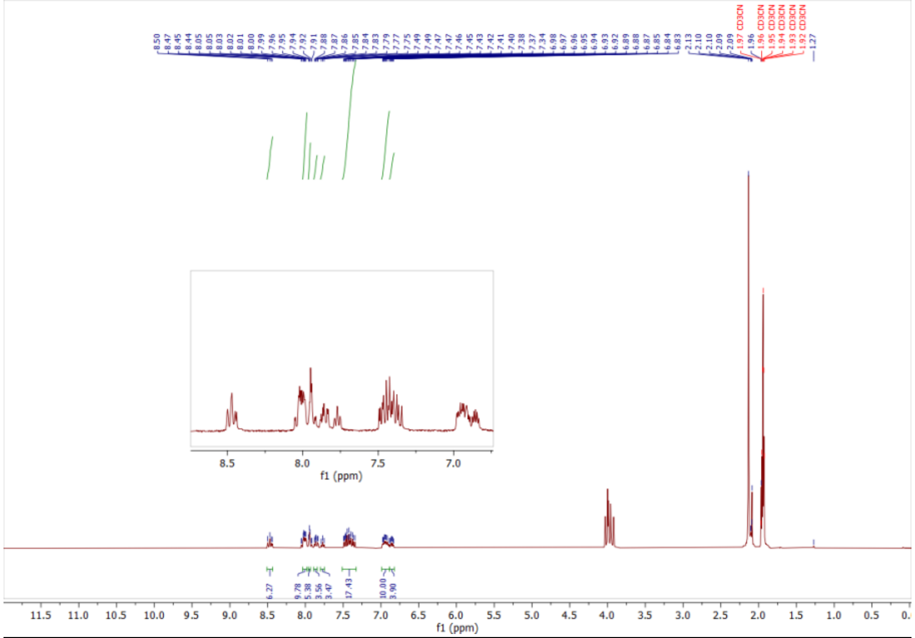

Figure S3. ^{1}H-NMR spectrum of Ru-^{dMeO}bpy in CD_3CN.

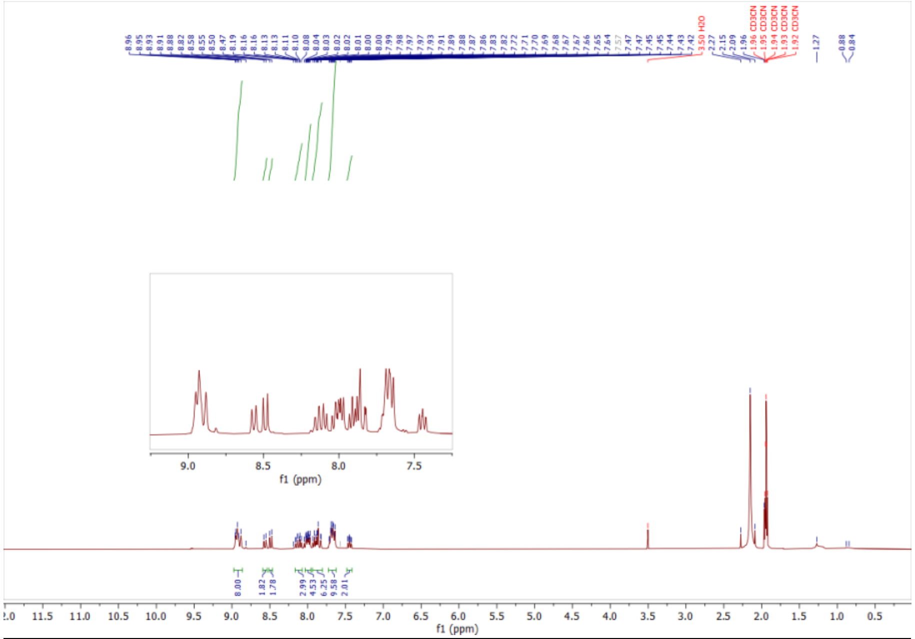

Figure S4. ^{1}H-NMR spectrum of Ru-^{dCF3}bpy in CD_3CN.

Spectroelectrochemistry

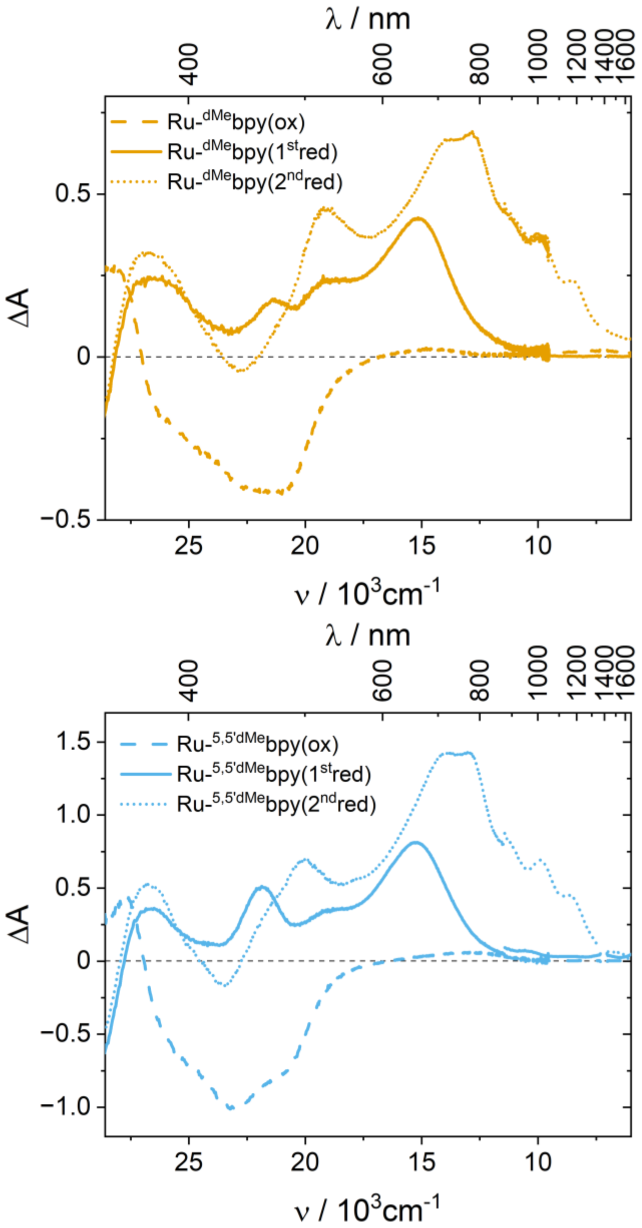

Figure S5. Differential spectra obtained upon oxidation (dashed), first reduction (solid) and second reduction (dotted) of Ru-^{dMe}bpy (top) and Ru-$^{5,5'dMe}$bpy (bottom) in acetonitrile with 100 mM $TBAPF_6$ as supporting electrolyte.

Steady-state Emission Spectroscopy

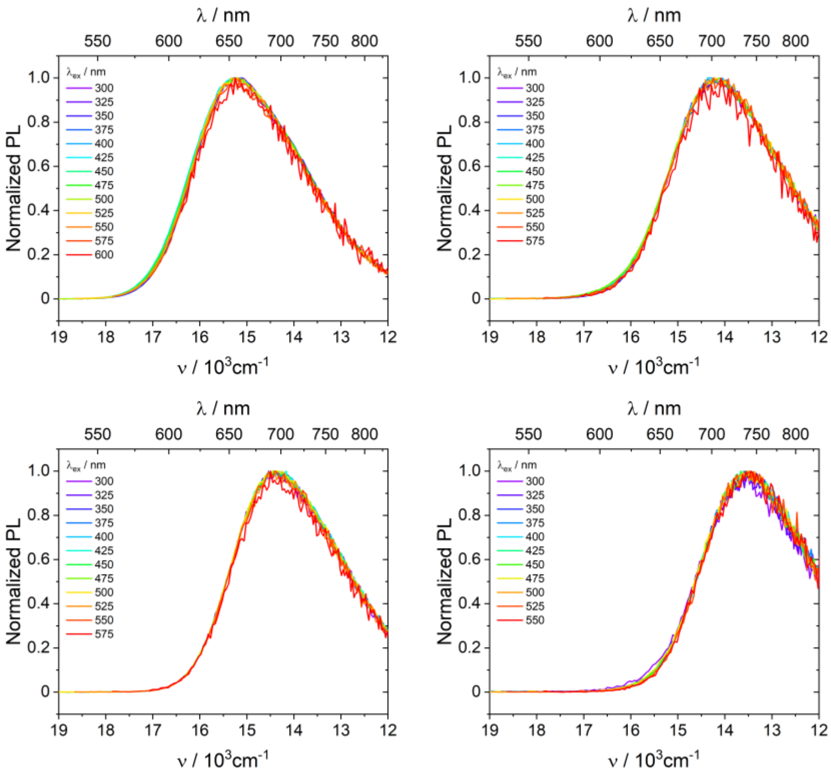

Figure S6. Steady-state emission spectra of Ru-^{dCF3}bpy (top left), Ru-^{dMe}bpy (top right), Ru-$^{5,5'-dMe}$bpy (bottom left) and Ru-^{dMeO}bpy (bottom right) in acetonitrile at room temperature, with different excitation wavelengths.

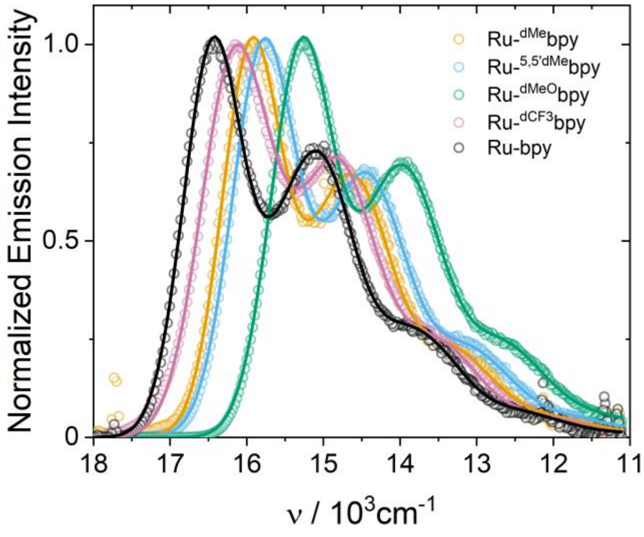

Figure S7. Steady-state emission spectra of Ru-bpy (black), Ru-^{dMe}bpy (orange), Ru-$^{5,5'-dMe}$bpy (cyan), Ru-^{dMeO}bpy (green) and Ru-^{dCF3}bpy (magenta) in butyronitrile at 77K (dots) and fits according to Franck-Condon Lineshape Analysis (solid curves).

Table S1. Photophysical parameters obtained upon Franck-Condon Lineshape Analysis of 80K emission spectra in butyronitrile.

Compound	$E_{0\text{-}0}$/ cm^{-1}	$\Delta\nu_{1/2}$/ cm^{-1}	S_1	$\hbar\omega_1$ / cm^{-1}	S_2	$\hbar\omega_2$ / cm^{-1}
**$[Ru(^{dCF3}bpy)_3]^{2+}$ [a]**	16830	620	0.84	1420	0.87	480
**Ru-^{dCF3}bpy**	16184	1011	0.88	1387	0.32	680
**$[Ru(bpy)_3]^{2+}$ [a]**	17400	605	0.98	1390	1.08	440
**Ru-bpy**	16439	907	0.75	1489	0.47	943
**$[Ru(^{dMe}bpy)_3]^{2+}$ [a]**	16940	790	1.08	1380	0.81	470
**Ru-^{dMe}bpy**	15931	913	0.64	1487	0.49	960
**$[Ru(^{5,5'dMe}bpy)_3]^{2+}$ [a]**	17540	750	0.87	1430	0.54	680
**Ru-$^{5,5'dMe}$bpy**	15775	975	0.61	1545	0.50	1049
**$[Ru(^{dMeO}bpy)_3]^{2+}$ [a]**	16470	850	1.05	1390	0.80	470
**Ru-^{dMeO}bpy**	15275	965	0.63	1513	0.51	1017

[a] Extracted from reference [10]

Time-Resolved Emission Spectroscopy

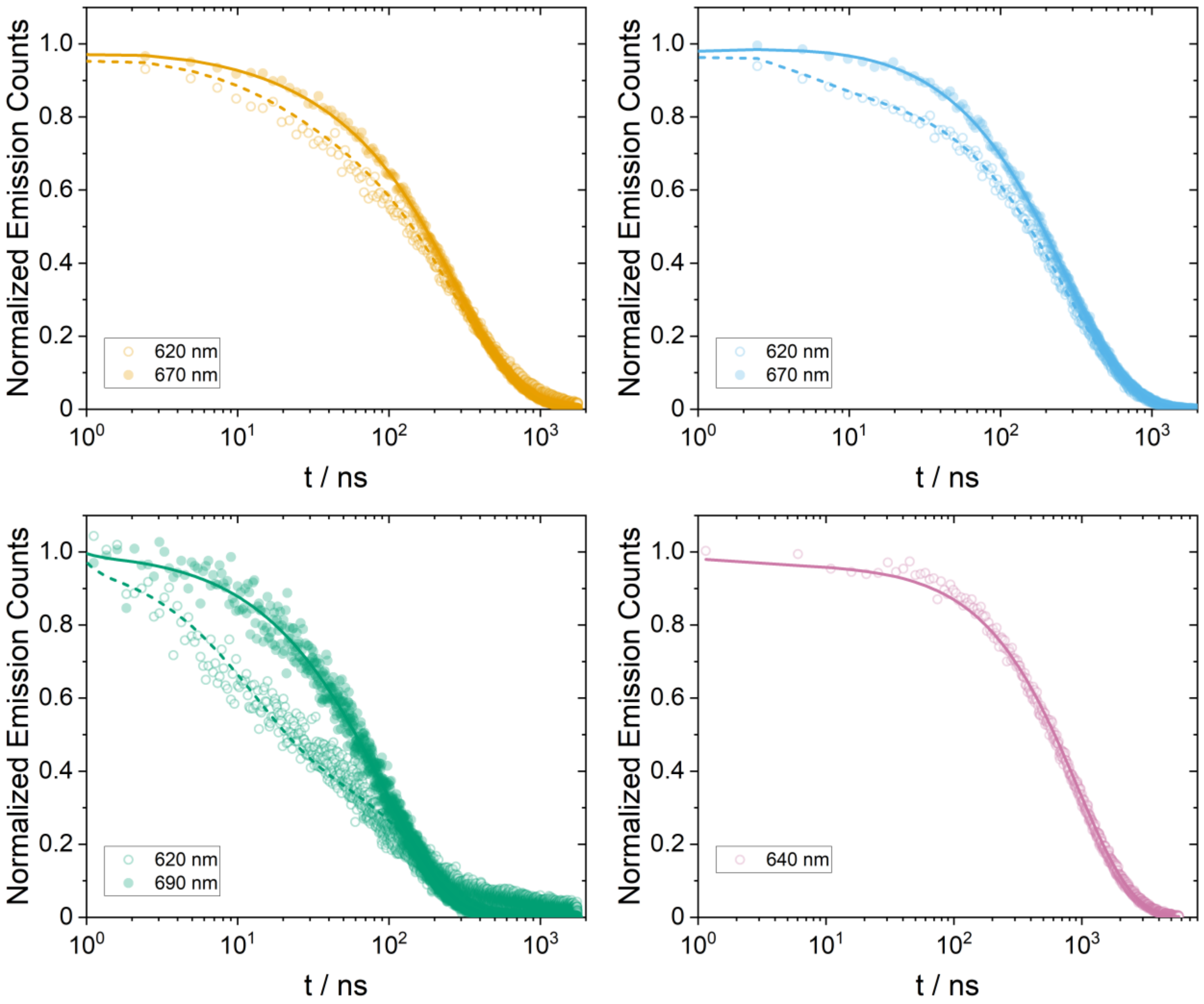

Figure S8. Emission decay traces of of Ru-^{dMe}bpy (orange, top left), Ru-$^{5,5'-dMe}$bpy (cyan, top right), Ru-^{dMeO}bpy (green, bottom left) and Ru-^{dCF3}bpy (magenta, bottom right) in acetonitrile at room temperature (dots) and fits.

Nanosecond Transient Absorption Spectroscopy

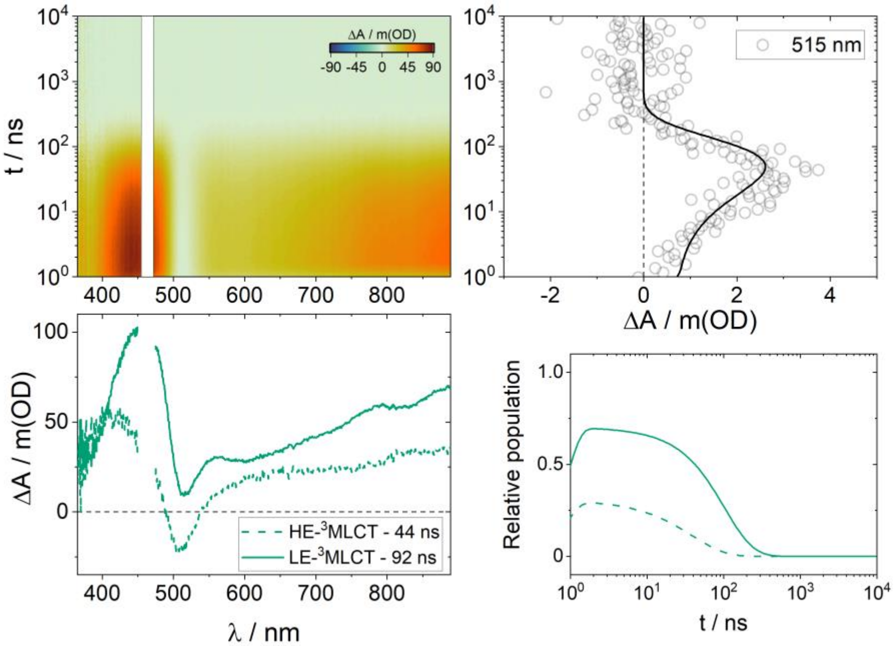

Figure S9. nsTAS map of Ru-^{dMeO}bpy in acetonitrile upon 460 nm excitation at room temperature (top left), kinetic trace and fit at 515 nm (top right), species-associated differential spectrum for HE-^{3}MLCT (dashed) and LE-^{3}MLCT (solid) (bottom left), and relative population evolution (bottom right).

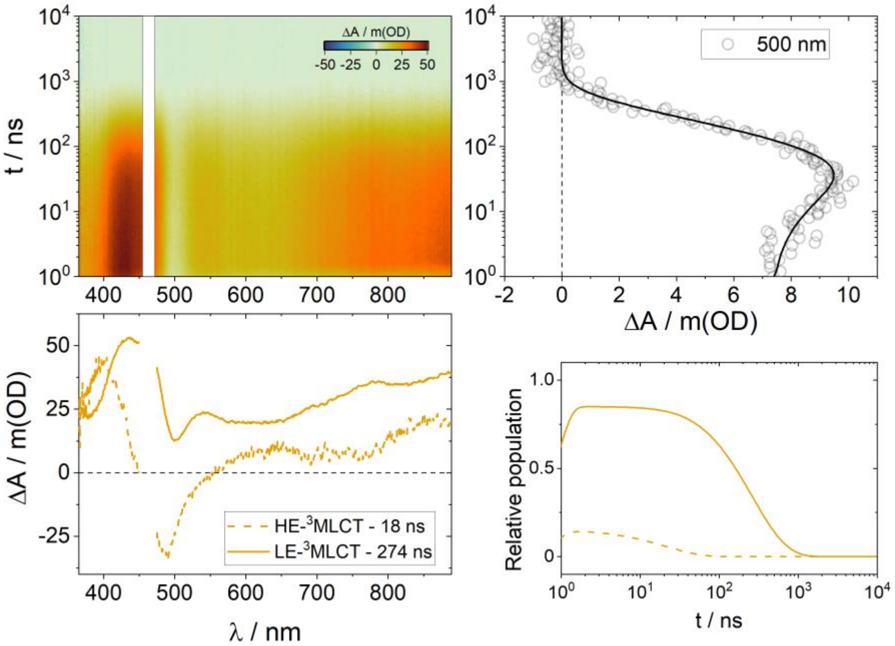

Figure S10. nsTAS map of Ru-^{dMe}bpy in acetonitrile upon 460 nm excitation at room temperature (top left), kinetic trace and fit at 500 nm (top right), species-associated differential spectrum for HE-^{3}MLCT (dashed) and LE-^{3}MLCT (solid) (bottom left), and relative population evolution (bottom right).

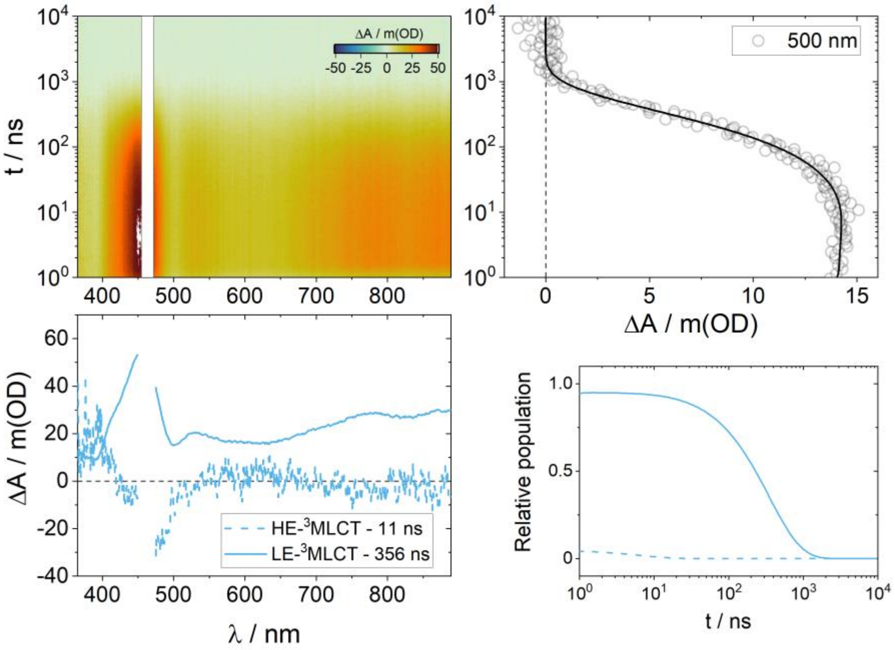

Figure S11. nsTAS map of Ru-$^{5,5'dMe}$bpy in acetonitrile upon 460 nm excitation at room temperature (top left), kinetic trace and fit at 500 nm (top right), species-associated differential spectrum for HE-^{3}MLCT (dashed) and LE-^{3}MLCT (solid) (bottom left), and relative population evolution (bottom right).

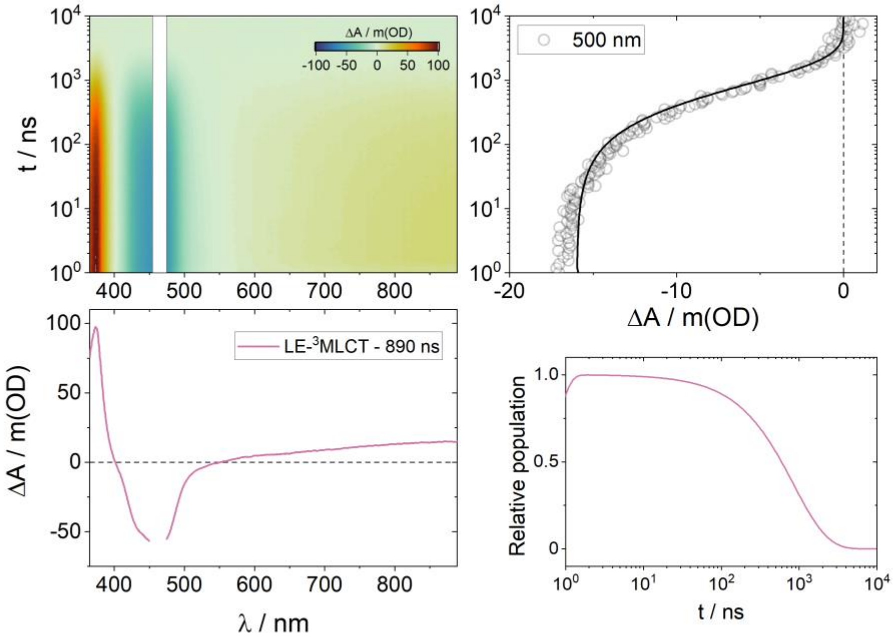

Figure S12. nsTAS map of Ru-^{dCF3}bpy in acetonitrile upon 460 nm excitation at room temperature (top left), kinetic trace and fit at 500 nm (top right), species-associated differential spectrum for LE-^{3}MLCT (bottom left), and relative population evolution (bottom right).

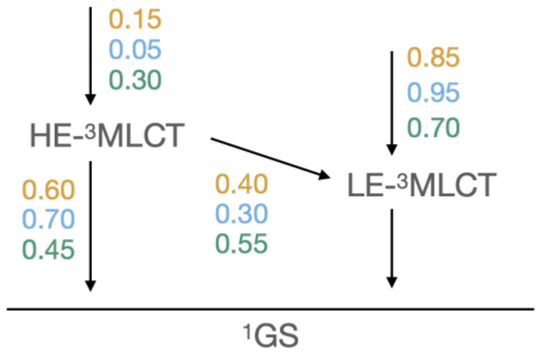

Figure S13. Target models used to fit nsTAS data, with the branching parameters for Ru-^{dMe}bpy (orange), Ru-$^{5,5'\text{-}dMe}$bpy (cyan) and Ru-^{dMeO}bpy (green).

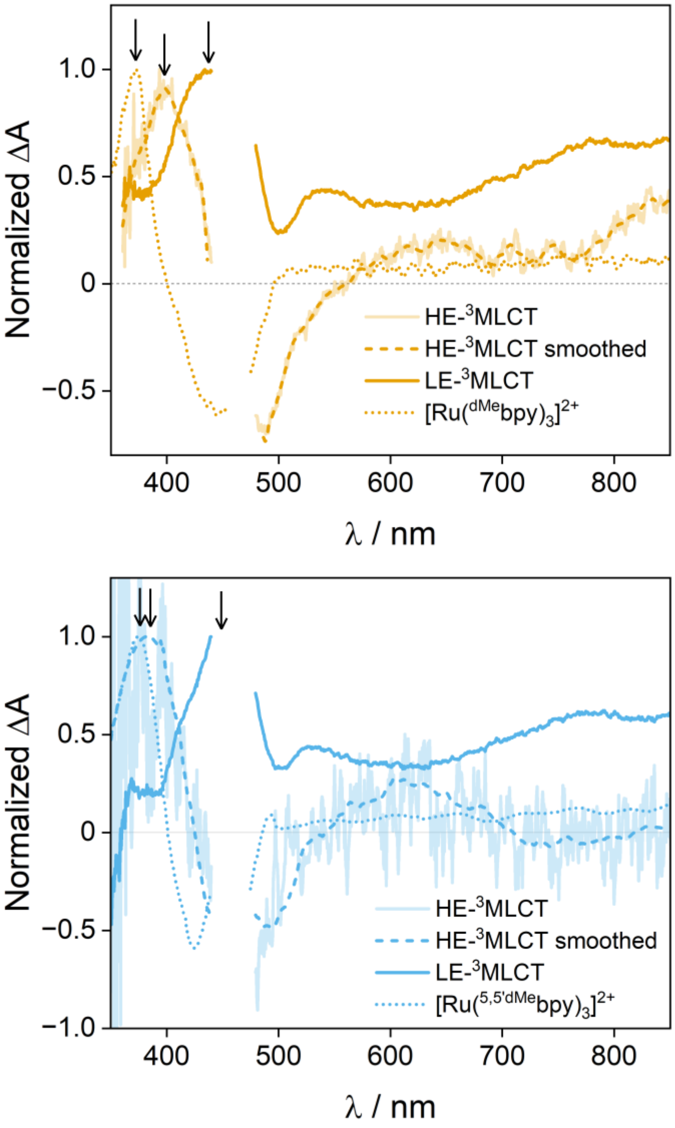

Figure S14. Species-associated differential spectra obtained upon nsTAS with 460 nm excitation of Ru-^{dMe}bpy (top) and Ru-$^{5,5'dMe}$bpy (bottom) and their respective monometallic references in acetonitrile at room temperature. Arrows indicate radical anion signals for each excited state. For HE-^{3}MLCT, smoothed spectra are also shown for clarity (see experimental details).

Femtosecond Transient Absorption Spectroscopy

For Ru-^{dMe}bpy, Ru-$^{5,5'dMe}$bpy and Ru-^{dMeO}bpy, our global-analysis modelling was based on three parallelly-decaying excited states, which afforded, next to their lifetimes, decay-associated differential spectra for each excited state. While the very short hot-HE'-^{3}MLCT(bpy) lifetimes relative to those of the corresponding monometallic references imply this state feeds via ILET the nanosecond-lived states, our attempts to employ models connecting them failed. However, the differential spectral shape of the resulting states compare very well with nsTAS and with the corresponding monometallic references, and therefore they are considered meaningful. For Ru-^{dMe}bpy, Ru-$^{5,5'dMe}$bpy and Ru-^{dCF3}bpy, lifetimes longer than 10 ns have been fixed to the values obtained in nsTAS. For Ru-^{dMeO}bpy, lifetimes longer than 10 ns have been fixed to the values obtained in TCSPC.

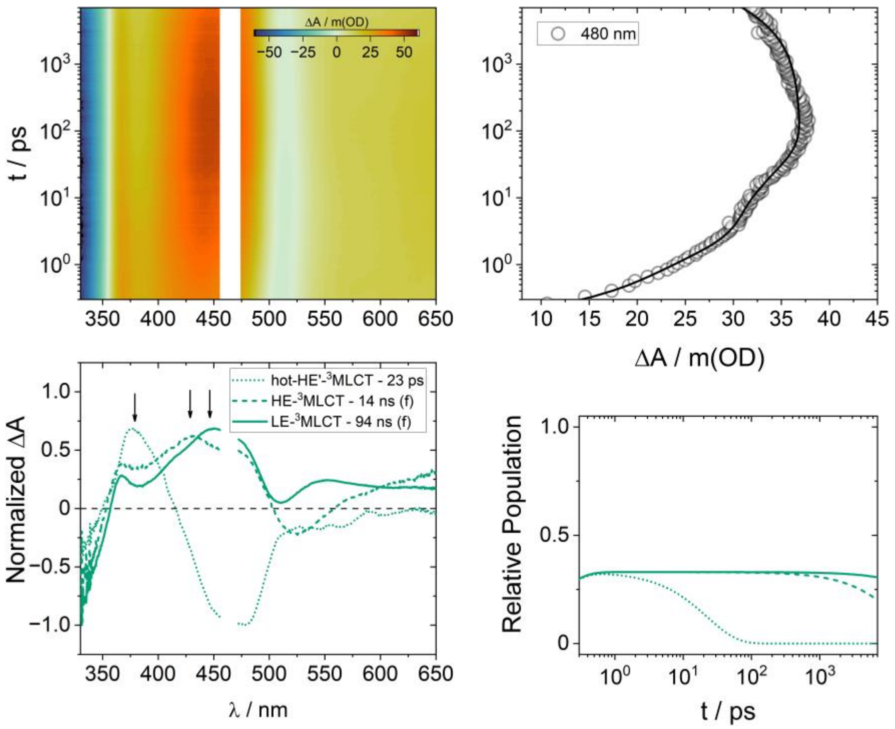

Figure S15. fsTAS map of Ru-^{dMeO}bpy in acetonitrile upon 460 nm excitation at room temperature (top left), kinetic trace and fit at 480 nm (top right), decay-associated differential spectrum for hot-HE'-^{3}MLCT (dotted), HE-^{3}MLCT (dashed), and LE-^{3}MLCT (solid) (bottom left), and relative population evolution (bottom right). Lifetimes longer than 10 ns have been fixed.

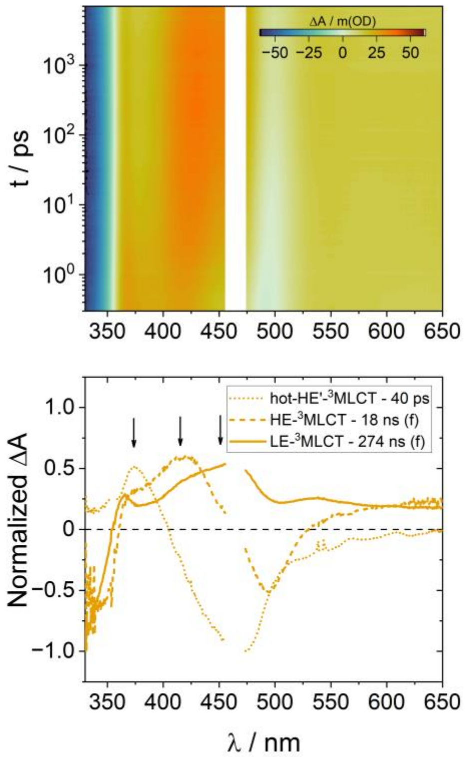

Figure S16. fsTAS map of Ru-^{dMe}bpy in acetonitrile upon 460 nm excitation at room temperature (top left), kinetic trace and fit at 480 nm (top right), decay-associated differential spectrum for hot-HE'-^{3}MLCT (dotted), HE-^{3}MLCT (dashed), and LE-^{3}MLCT (solid) (bottom left), and relative population evolution (bottom right). Lifetimes longer than 10 ns have been fixed.

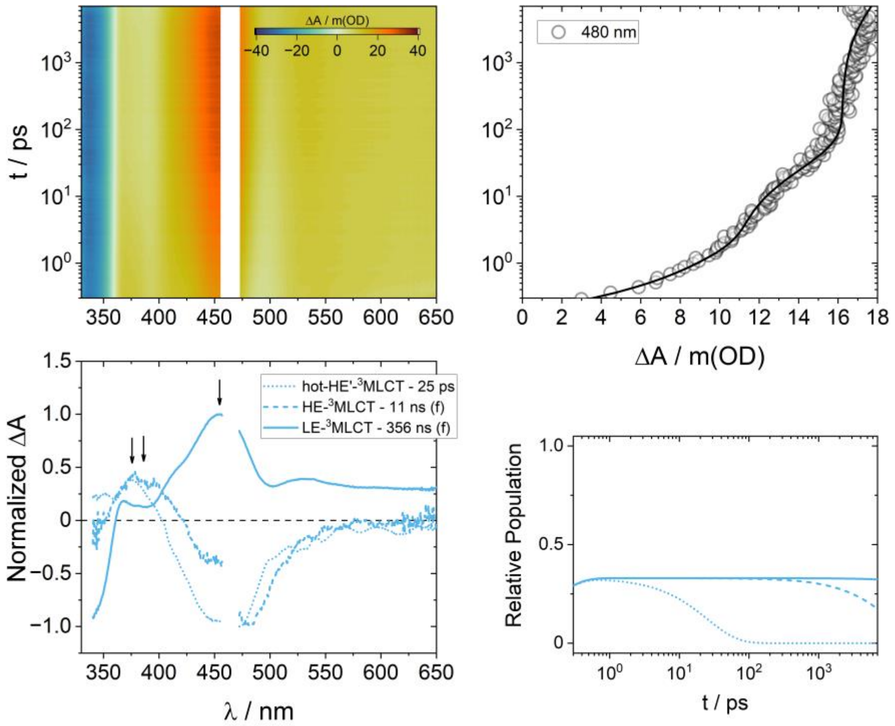

Figure S17. fsTAS map of Ru-$^{5,5'\mathrm{dMe}}$bpy in acetonitrile upon 460 nm excitation at room temperature (top left), kinetic trace and fit at 480 nm (top right), decay-associated differential spectrum for hot-HE'-^{3}MLCT (dotted), HE-^{3}MLCT (dashed), and LE-^{3}MLCT (solid) (bottom left), and relative population evolution (bottom right). Lifetimes longer than 10 ns have been fixed.

For Ru-^{dCF3}bpy, target analysis was employed using the model in **Figure S19**, affording physically meaningful species-associated differential spectra for each excited state.

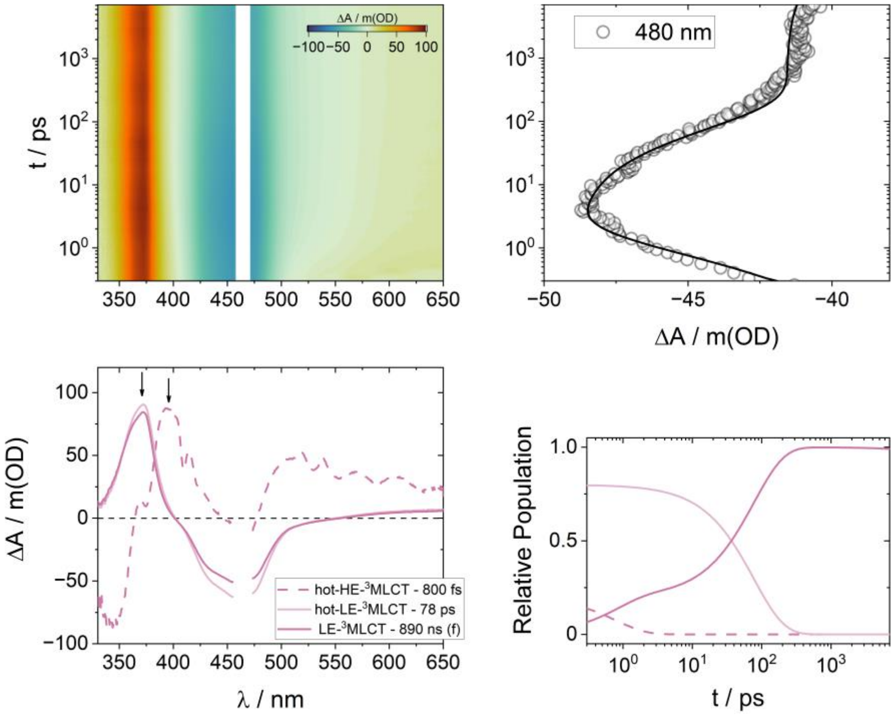

Figure S18. fsTAS map of Ru-^{dCF3}bpy in acetonitrile upon 460 nm excitation at room temperature (top left), kinetic trace and fit at 480 nm (top right), species-associated differential spectrum for hot-HE-^{3}MLCT (dashed), hot-LE-^{3}MLCT (solid, light), and LE-^{3}MLCT (solid, dark) (bottom left), and relative population evolution (bottom right). Lifetimes longer than 10 ns have been fixed.

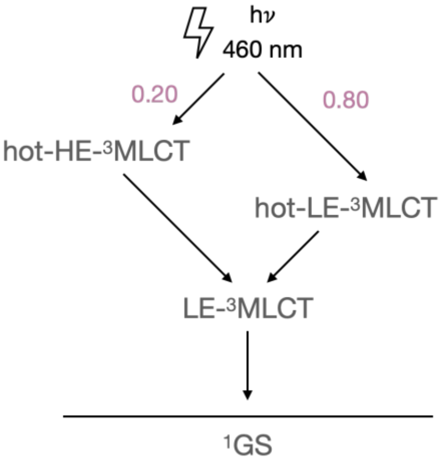

Figure S19. Target models used to fit fsTAS data, with the branching parameters for Ru-^{dCF3}bpy.

Stern-Volmer Studies

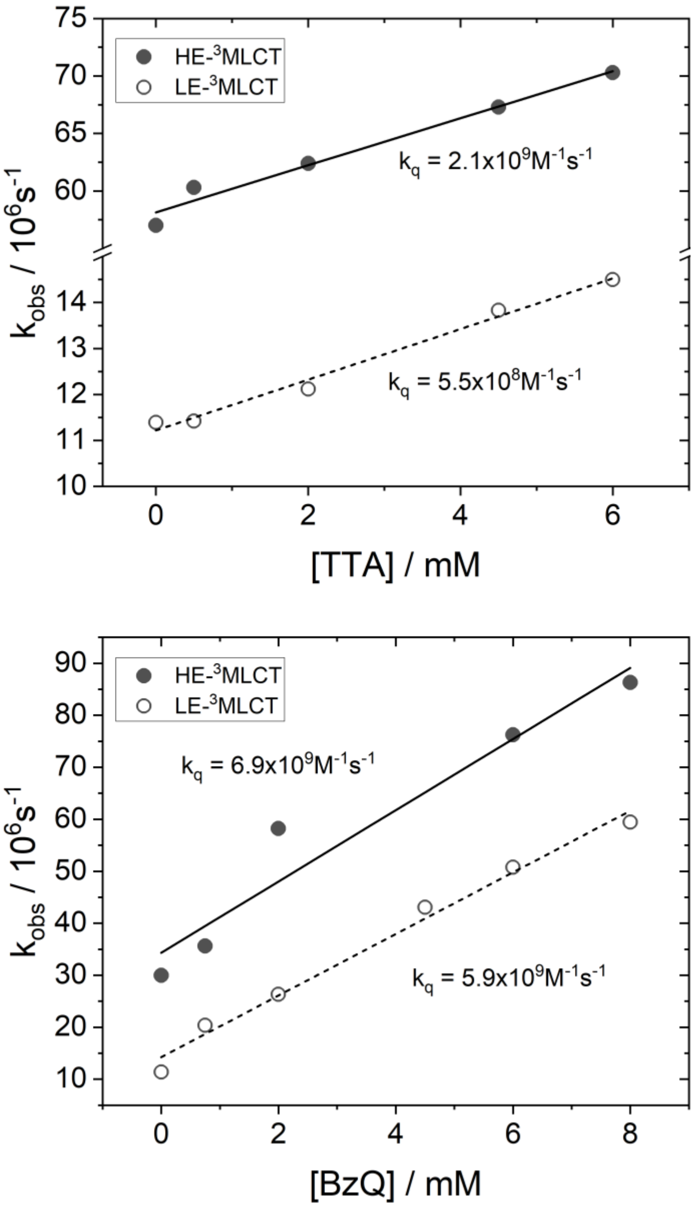

Figure S20. Stern-Volmer analysis upon nsTAS spectroscopy of Ru-^{dMeO}bpy (2 mM) upon 460 nm excitation, with TTA (top) and BzQ (bottom) in acetonitrile at room temperature.

References

[1] M. Krejčik, M. Daněk, F. Hartl, *J Electroanal Chem Interfacial Electrochem* **1991**, *317*, 179–187.

[2] W. R. Roberts, T. N. Rohrabaugh, R. M. O'Donnell, *Journal of Visualized Experiments* **2021**, 1–13.

[3] H. Ishida, S. Tobita, Y. Hasegawa, R. Katoh, K. Nozaki, *Coord Chem Rev* **2010**, *254*, 2449–2458.

[4] I. H. M. Van Stokkum, D. S. Larsen, R. Van Grondelle, *Biochim Biophys Acta Bioenerg* **2004**, *1657*, 82–104.

[5] J. J. Snellenburg, S. Laptenok, R. Seger, K. M. Mullen, I. H. M. van Stokkum, *J Stat Softw* **2012**, *49*, 1–22.

[6] K. M. Mullen, I. H. M. Van Stokkum, *J Stat Softw* **2007**, *18*, 1–46.

[7] B. Elias, S. Cerfontaine, L. Troian-Gautier, L. Troian-Gautier, S. A. M. Wehlin, F. Loiseau, E. Cauët, *Dalton Transactions* **2020**, *49*, 8096–8106.

[8] R. E. Rinehart, J. S. Lasky, *J Am Chem Soc* **1964**, *86*, 2516–2518.

[9] M. R. Norris, J. J. Concepcion, C. R. K. Glasson, Z. Fang, A. M. Lapides, D. L. Ashford, J. L. Templeton, T. J. Meyer, *Inorg Chem* **2013**, *52*, 12492–12501.

[10] F. Glaser, S. De Kreijger, K. Achilleos, L. Narayan Satheesh, A. Ripak, N. Chantry, C. Bourgois, S. Quiquempoix, J. Scriven, J. Rubens, M. Vander Wee-Léonard, M. Daenen, M. Gillard, B. Elias, L. Troian-Gautier, *ChemPhotoChem* **2024**, DOI 10.1002/cptc.202400134.
